# Lactucin Synthase Inactivation Boosts the Accumulation of Anti-inflammatory 8-Deoxylactucin and Its Derivatives in Chicory (*Cichorium intybus* L.)

**DOI:** 10.1021/acs.jafc.2c08959

**Published:** 2023-04-10

**Authors:** Katarina Cankar, Johanna Christina Hakkert, Robert Sevenier, Christina Papastolopoulou, Bert Schipper, João P. Baixinho, Naiara Fernández, Melanie S. Matos, Ana Teresa Serra, Claudia Nunes Santos, Khabat Vahabi, Alain Tissier, Paul Bundock, Dirk Bosch

**Affiliations:** † Wageningen Plant Research, Wageningen University & Research, Droevendaalsesteeg 1, 6708PB Wageningen, The Netherlands; ‡ Keygene N.V., Agro Business Park 90, 6708PW Wageningen, Netherlands; § Instituto de Biologia Experimental e Tecnológica (iBET), Av. República, Qta. Marquês, 2780-157 Oeiras, Portugal; ∥ Instituto de Tecnologia Química e Biológica António Xavier, Universidade Nova de Lisboa (ITQB NOVA), Av. da República, 2780-157 Oeiras, Portugal; ⊥ iNOVA4Health, NOVA Medical School Faculdade de Ciências Médicas, NMS|FCM, Universidade Nova de Lisboa, 1169-056 Lisboa, Portugal; # Department of Cell and Metabolic Biology, Leibniz Institute of Plant Biochemistry, 06120 Halle (Saale), Germany; ¶ Martin-Luther-Universität Halle-Wittenberg, Institut für Pharmazie, Kurt-Mothes-Str. 3, 06120 Halle (Saale), Germany

**Keywords:** chicory, sesquiterpene lactones, lactucin synthase, genome editing, CRISPR/Cas9, 8-deoxylactucin, anti-inflammatory activity

## Abstract

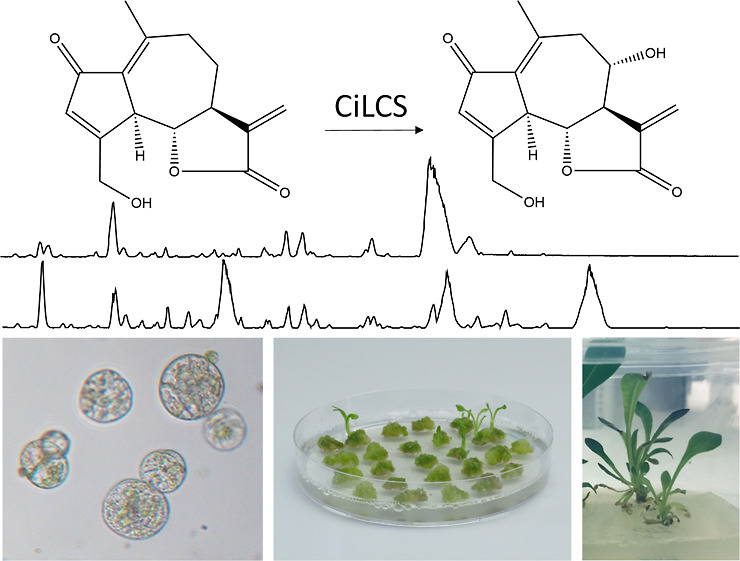

For several sesquiterpene lactones (STLs) found in Asteraceae plants, very interesting biomedical activities have been demonstrated. Chicory roots accumulate the guaianolide STLs 8-deoxylactucin, lactucin, and lactucopicrin predominantly in oxalated forms in the latex. In this work, a supercritical fluid extract fraction of chicory STLs containing 8-deoxylactucin and 11β,13-dihydro-8-deoxylactucin was shown to have anti-inflammatory activity in an inflamed intestinal mucosa model. To increase the accumulation of these two compounds in chicory taproots, the lactucin synthase that takes 8-deoxylactucin as the substrate for the regiospecific hydroxylation to generate lactucin needs to be inactivated. Three candidate cytochrome P450 enzymes of the CYP71 clan were identified in chicory. Their targeted inactivation using the CRISPR/Cas9 approach identified CYP71DD33 to have lactucin synthase activity. The analysis of the terpene profile of the taproots of plants with edits in *CYP71DD33* revealed a nearly complete elimination of the endogenous chicory STLs lactucin and lactucopicrin and their corresponding oxalates. Indeed, in the same lines, the interruption of biosynthesis resulted in a strong increase of 8-deoxylactucin and its derivatives. The enzyme activity of CYP71DD33 to convert 8-deoxylactucin to lactucin was additionally demonstrated *in vitro* using yeast microsome assays. The identified chicory lactucin synthase gene is predominantly expressed in the chicory latex, indicating that the late steps in the STL biosynthesis take place in the latex. This study contributes to further elucidation of the STL pathway in chicory and shows that root chicory can be positioned as a crop from which different health products can be extracted.

## Introduction

**Figure 1:**
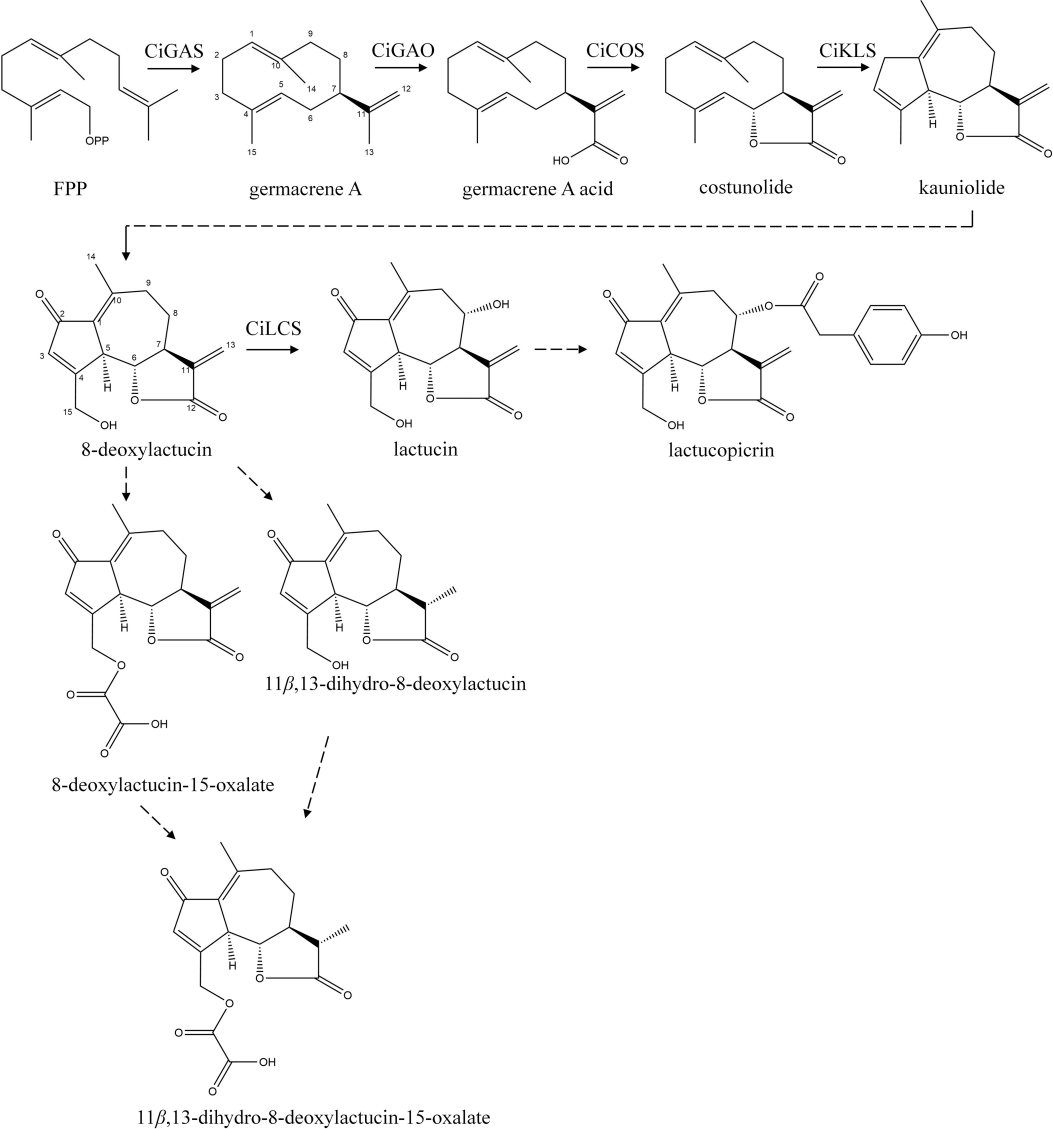
Putative STL biosynthetic pathway in chicory. The biosynthetic pathway of chicory STLs has been partly elucidated. Enzymes catalyzing the conversion of FPP to kauniolide have been previously characterized. The chicory lactucin synthase CiLCS converts 8-deoxylactucin to lactucin (this work). Dashed arrows indicate uncharacterized enzymatic conversions. FPP—farnesyl pyrophosphate, CiGAS—germacrene A synthase, CiGAO—germacrene A oxidase, CiCOS—costunolide synthase, CiKLS—kauniolide synthase, and CiLCS—lactucin synthase.

*Cichorium intybus* var. *sativum* or root chicory is currently cultivated predominantly in northern France, Belgium, and the Netherlands for the extraction of the dietary fiber inulin, which is used as a prebiotic and sweetener.^1,2^ Besides inulin, chicory roots are rich in bitter sesquiterpene lactones (STLs), which accumulate in the latex. In chicory, three major STLs, namely 8-deoxylactucin, lactucin, and lactucopicrin, accumulate, predominantly in the oxalated form^3^ (Figure 1). The accumulation of lactucin and lactucopicrin has also been described in other Asteraceae plants, such as lettuce (*Lactuca sativa*) and wild lettuce (*Lactuca virosa*).^3^

There are several reports describing bioactivities for the guaianolide STLs from chicory. Lactucin and lactucopicrin, in particular, have been described to have analgesic and sedative effects in mice^4^ and to have antimalarial activity.^5^ It has also been shown that natural extracts obtained from chicory roots present antibacterial and antifungal properties.^6^ Transcription factors and other molecular players involved in pro-inflammatory signaling are among the biological targets upon which STLs are known to exert their anti-inflammatory bioactivity, and although extensive studies can already be found on this class of chemicals, the reports on chicory STLs are still scarce.^7^ Recently, 11β,13-dihydrolactucin, a lactucin derivative found in chicory, has revealed a promising anti-inflammatory potential in an yeast inflammatory model.^8^ Chicory STLs 11β,13-dihydrolactucin, lactucin, 11β,13-dihydro-8-deoxylactucin, 8-deoxylactucin, 11β,13-dihydrolactucopicrin, and lactucopicrin have been isolated from chicory roots by supercritical CO_2_ extraction. A subsequent chromatography fractionation yielded a fraction containing a mixture of 8-deoxylactucin and 11β,13-dihydro-8-deoxylactucin, which presented a promising anti-inflammatory activity as shown in the same yeast reporter system.^9^ The current understanding on the mode of action of STLs to fight inflammation has recently been reviewed.^7^

The biosynthetic pathway leading to chicory STLs has been partially elucidated (Figure 1). The common terpene precursor FPP is first cyclized to germacrene A by the germacrene A synthase (CiGAS),^10^ and consequently, three cytochrome P450 enzymes, namely the germacrene A oxidase (CiGAO),^11^ costunolide synthase (CiCOS),^12,13^ and kauniolide synthase (CiKLS)^14,15^ catalyze the next steps in the STL biosynthesis to form kauniolide, with a specific tricyclic structure typical of guaianolide STLs. The following steps in the biosynthesis have not been elucidated but presumably involve further oxygenation of kauniolide on positions 2 and 15 by cytochrome P450 enzymes to yield 8-deoxylactucin. The downstream enzymes that catalyze the conversion of 8-deoxylactucin to lactucin and lactucopicrin are also not yet elucidated. Because cytochrome P450 oxygenases of the CYP71 clan are frequently involved in the oxygenation of STLs in Asteraceae^16−21^ and the enzymes GAO, COS, and KLS catalyzing initial steps of chicory STL biosynthesis also belong to the CYP71 clan,^11−15^ it is likely that a CYP71 enzyme carries out the hydroxylation of 8-deoxylactucin at the 8 position to generate lactucin. Next, the hydroxy group on the 8-position of lactucin is conjugated to hydroxyphenyl acetic acid to form lactucopicrin. The majority of the STLs in chicory are further conjugated to oxalate at position 15. 8-deoxylactucin itself is converted via oxalate transfer and via reduction of the double bond between positions 11 and 13 into its derivatives 8-deoxylactucin-15-oxalate, 11β,13-dihydro-8-deoxylactucin, and 11β,13-dihydro-8-deoxylactucin-15-oxalate, as illustrated in Figure 1. Enzymes involved in oxalate transfer and reduction of the double bond have not yet been identified. The later steps in the STL biosynthesis, from kauniolide onward, appear to take place in the latex itself, as the gene encoding kauniolide synthase has latex-specific expression.^14^

Several protocols for CRISPR/Cas9-based genome editing have been established for chicory.^22−24^ Enzymes in the terpene biosynthetic pathway have been successfully inactivated in chicory using the CRISPR/Cas9 approach. The germacrene A synthase *CiGAS* is present in the chicory genome in four copies,^25^ and its inactivation led to the elimination of STL biosynthesis.^23^ Inactivation of the three kauniolide synthase genes (*CiKLS*) in chicory led to the accumulation of costunolide and its conjugates in chicory taproots.^14^

In this work, we describe the identification of the lactucin synthase in chicory and demonstrate that inactivation of the corresponding gene by CRISPR/Cas genome editing results in significant accumulation of 8-deoxylactucin and its derivatives that have anti-inflammatory properties in the taproot.

## Results

### SFE Fraction Containing 8-Deoxylactucin and 11β,13-Dihydro-8-deoxylactucin Shows Anti-inflammatory Activity in an Inflamed Intestinal Mucosa Model

**Figure 2:**
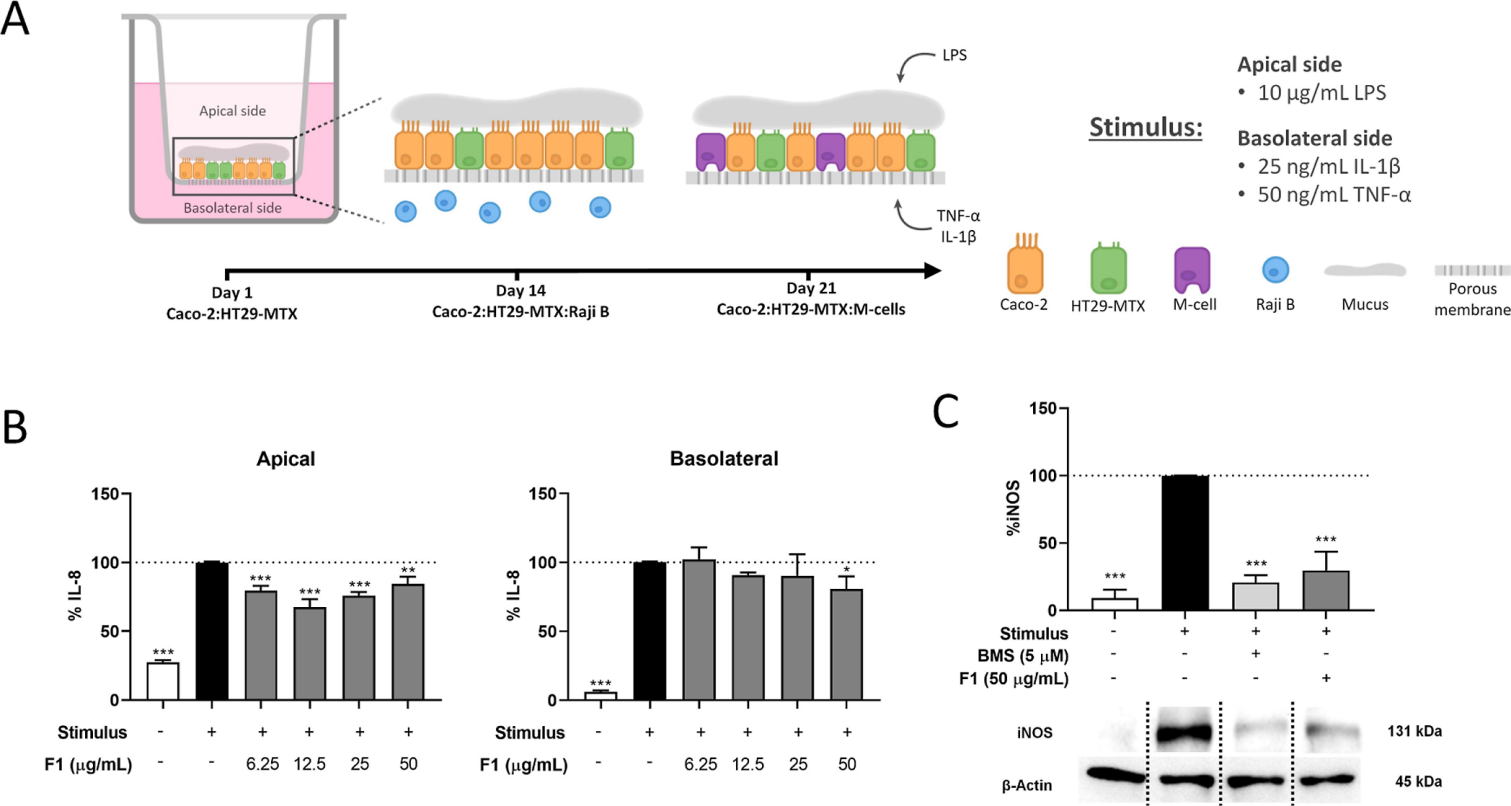
8-deoxylactucin/11β,13-dihydro-8-deoxylactucin-rich SFE fraction (fraction 1, F1) displays the anti-inflammatory effect in Caco-2/HT29-MTX/RajiB co-culture. (A) Triple cell co-culture experimental setup of the inflamed intestinal mucosa (10 μg/mL of LPS on the apical side; 25 ng/mL of IL-1β, and 50 ng/mL of TNF-α on the basolateral side). (B) IL-8 release assessed by ELISA in both apical and basolateral supernatants of cells treated with a range of concentrations of F1 for 48 h in co-incubation with the pro-inflammatory stimulus. (C) iNOS expression levels assessed by Western blot after treatment of cells with an IKK-1/IKK-2 inhibitor (BMS 345541) or F1 for 12 h in co-incubation with the pro-inflammatory stimulus. All results are presented as a percentage of the stimulated control and were obtained from at least three independent biological replicates. *p < 0.05, **p < 0.01, ***p < 0.001 relative to the stimulated control.

An intestinal triple co-culture model (Figure 2A) composed of Caco-2/HT29-MTX/RajiB cells challenged with a cocktail of pro-inflammatory stimulus (Figure S1) was used to evaluate the anti-inflammatory potential of non-cytotoxic concentrations of the chicory supercritical fluid extract (SFE) and three purified SFE fractions (Figure S2). The three fractions obtained are enriched in different compounds (Fraction 1, F1: 8-deoxylactucin and 11β,13-dihydro-8-deoxylactucin; Fraction 2, F2: 11β,13-dihydrolactucopicrin and lactucopicrin; Fraction 3, F3: 11β,13-dihydrolactucin and lactucin), as previously described^9^ (Figure S2). SFE and F1 were the only samples able to significantly decrease IL-8 release in both apical and basolateral sides of the intestinal mucosa model, which depict local and systemic effects, respectively, although F1 efficacy is observed at lower concentrations than SFE. To confirm the results, combinations of pure STLs were tested to mimic fraction compositions (lactucopicrin + 11β,13-dihydrolactucopicrin and lactucin + 11β,13-dihydrolactucin) in the same concentrations as those present in fraction 2 and fraction 3, respectively (Figure S2). However, this approach was not feasible for fraction F1 since neither 8-deoxylactucin nor 11β,13-dihydro-8-deoxylactucin are commercially available. It should be noted that fraction F1 was the least cytotoxic of the three, allowing it to be tested at a concentration 10-fold higher than that of F2 or F3 (50 μg/mL for F1 vs 5 μg/mL for F2 or F3), which may explain its higher efficacy in decreasing IL-8 release from cells submitted to an inflammatory insult (Figure S2). As 100 μg/mL already starts to decrease cell viability (Figure S2A), the concentration of 50 μg/mL was considered the highest concentration to be applied in the assay without compromising cell viability. This result demonstrates the potential ability of F1 to modulate intestinal inflammation.

As F1 was found to be the most promising fraction purified from SFE, a dose–response assay was conducted with four concentrations of this sample (Figure 2B), ranging from 6.25 to 50 μg/mL, that were added to the apical chamber in a 48 h co-incubation with the pro-inflammatory stimulus. IL-8 levels were measured in both apical and basolateral supernatants, which revealed different results. All concentrations of F1 were able to significantly decrease IL-8 release to the apical compartment, revealing a promising local anti-inflammatory effect, whereas only the highest concentration (50 μg/mL) could significantly reduce IL-8 levels in the basolateral compartment, suggesting the need for a higher concentration of fractions in order to achieve systemic effects.

To further assess the anti-inflammatory potential of F1, the inducible nitric oxide synthase (iNOS) protein expression described to be induced by an inflammatory stimulus was studied by Western blot (Figure 2C). For this purpose, co-cultured cells were co-incubated with F1 and the pro-inflammatory stimulus for 12 h before protein collection and determination. Results showed that 50 μg/mL of F1 significantly reduced the expression of iNOS to levels close to those achieved by BMS 345541, an IKK-1/IKK-2 inhibitor used as the positive control (30% vs 21% iNOS expression when compared to the inflamed untreated control).

### Identification of the Candidate Genes Involved in STL Biosynthesis

To preferentially accumulate 8-deoxylactucin and its derivatives in chicory roots, the enzyme that converts 8-deoxylactucin to lactucin needs to be inactivated. This enzyme catalyzes a regiospecific hydroxylation of 8-deoxylactucin on the 8 position and presumably belongs to cytochrome P450 enzymes. The chicory genome of variety Orchies used in this study (NCBI BioProject PRJNA901408) was examined to identify putative cytochrome P450s of clan CYP71, and root tissue transcriptome data of the same chicory variety (NCBI BioProject PRJNA824299) were used to yield a subselection of these enzymes, which are expressed in chicory taproots. By this approach, three cytochrome P450s were identified that cluster with the parthenolide synthase from feverfew (TpPTS)^21^ and the eupatolide synthase from sunflower (HaES),^17^ which are involved in the STL biosynthesis in these Asteraceae species (Figure S3A). Based on these observations, it was postulated that the three identified cytochrome P450s may play a role in the biosynthesis of chicory STLs. The identified enzymes were classified as belonging to the clan CYP71 of cytochrome P450 enzymes^26^ and were assigned CYP names CYP71DD20, CYP71DD33 and CYP71DD35. The cDNA and protein sequences of these genes were submitted to NCBI under accession numbers OP973198, OP973199 and OP973200. The amino acid sequence alignment of the three enzymes to TpPTS and HaES, indicating the conserved motifs of plant cytochrome P450s^27^ is shown in Figure S3B. CYP71DD33 shows 73 and 56% amino acid identity to CYP71DD35 and CYP71DD20, respectively (Figure S3C). CYP71DD35 and CYP71DD20 show 56% amino acid identity to each other. When comparing to previously characterized cytochrome P450 enzymes that were described to catalyze STL biosynthesis in Asteraceae species CYP71DD33 and CYP71DD35 show the closest amino acid identity to TpPTS both at 54%, while CYP71DD20 shows the closest amino acid identity to HaES at 55%.

**Figure 3:**
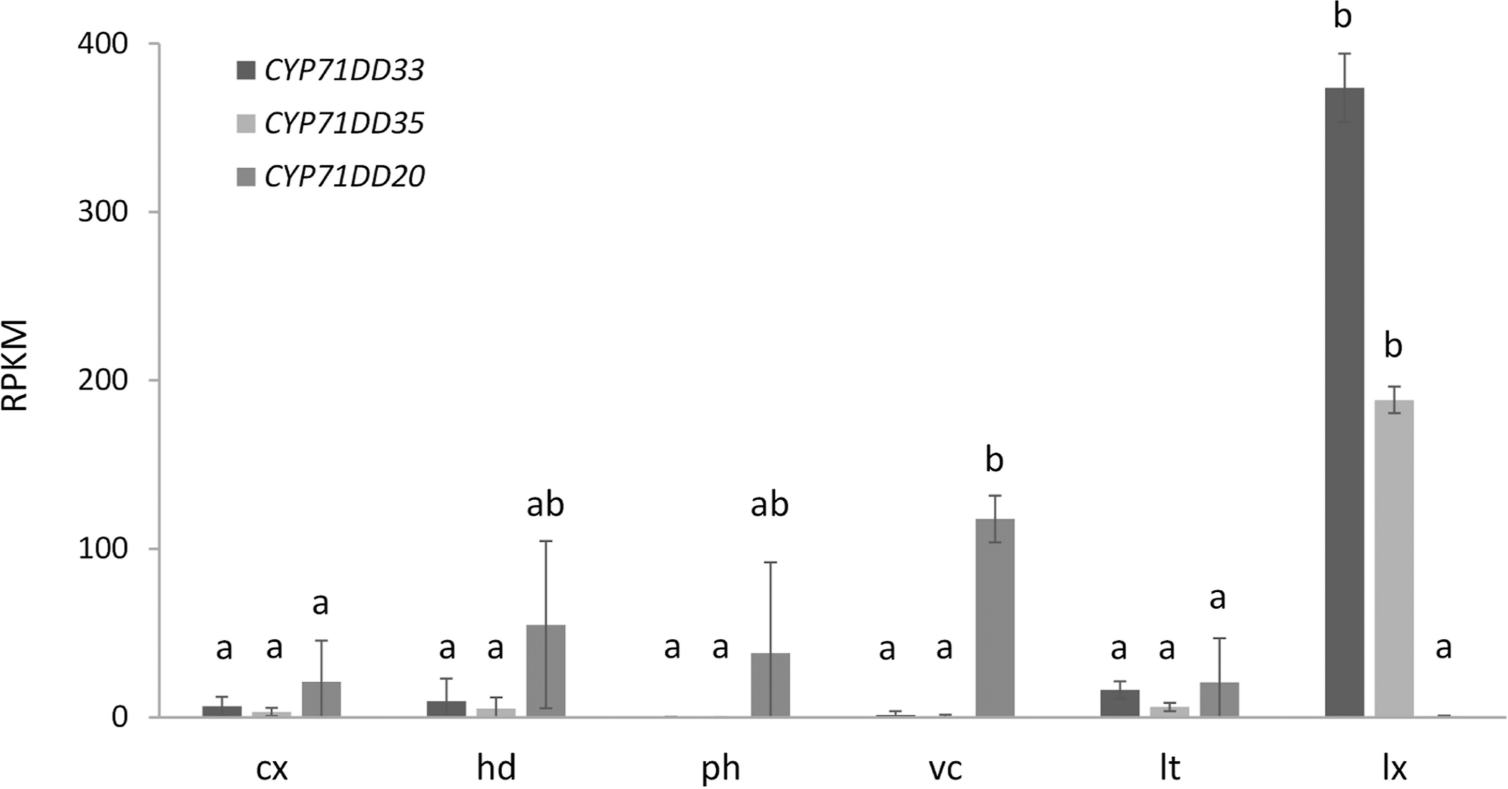
Gene expression analysis of *CYP71DD33*, *CYP71DD35,* and *CYP71DD20* in chicory taproot tissues. Gene expression is shown for different taproot tissues: cx—cortex, hd—hypodermis, ph—phloem, vc—vascular cylinder, lt—laticifer, lx—latex. RPKM—Reads per kilo base per million mapped reads. The letters indicate significantly different groups according to ANOVA and the Tukey’s post hoc test (*P* < 0.05).

Next, the gene expression of *CYP71DD33*, *CYP71DD35,* and *CYP71DD20* was examined in different tissues of the chicory taproot. Both *CYP71DD33* and *CYP71DD35* show the strongest expression in the chicory latex and very low expression in other root tissues (Figure 3). The expression of *CYP71DD33* in latex was twofold higher than the expression of gene *CYP71DD35*. On the contrary, *CYP71DD20* shows expression in several root tissues, with the strongest expression in the vascular cylinder.

### Inactivation of the Candidate Cytochrome P450 Genes in Chicory by CRISPR/Cas9

To study whether CYP71DD33, CYP71DD35, and CYP71DD20 indeed catalyze biosynthetic steps of chicory STL biosynthesis, the corresponding genes were next inactivated in chicory using CRISPR/Cas9-based genome editing. The CRISPR/Cas9 reagents were delivered to chicory protoplasts via transient transfection of two plasmids encoding the Cas9 protein and guide RNAs for *CYP71DD33* or via transient transfection of the ribonucleoprotein complexes (RNPs) for *CYP71DD35* and *CYP71DD20*, as previously described.^14,23^

To inactivate *CYP71DD33,* four guide RNAs were designed to target exon 1 of the gene (Table S1). Guides were designed within a 120 bp region to enable efficient genotyping. The initial genotyping of the *CYP71DD33* genome-edited lines was performed at an early stage during the plant regeneration. To this end, a section of exon 1 of *CYP71DD33* was amplified, the PCR products were sequenced by Sanger sequencing, and the sequences were examined for mutations. By this approach, quick screening and selection of genome-edited chicory lines were achieved; however, the precise nature of the mutations in the two alleles was not determined at this stage. The editing frequency observed in calli and shoots was 21 and 22%, respectively (Table 1). In total, 19 lines with mutations in *CYP71DD33* were obtained by this approach. Detailed genotyping was performed for eight regenerated genome-edited lines (Table S2). The analysis revealed that efficient editing was obtained by three of the designed guide RNAs, which resulted in deletions ranging from 1 bp up to 102 bp. Often, the region between two guides was deleted. The insertion events were more rare. We observed a single nucleotide insertion in line 64. In line 53, a large insertion of 125 base pairs was observed in one of the alleles. For five chicory lines, biallelic mutations were confirmed. For lines 26, 57, and 64, the analysis of 7 to 11 cloned PCR products revealed the same mutations. Therefore, these lines seem to carry homozygous mutations. In none of the analyzed lines, WT alleles were observed, indicating that indeed both alleles of *CYP71DD33* were efficiently edited in the eight regenerated chicory lines. As it was previously shown that plasmid-based genome editing may lead to *Cas9* gene integration in the genome,^14,22,23^ we have analyzed eight regenerated chicory lines with mutations in *CYP71DD33* by PCR using primers specific for the *Cas9* gene. Indeed, *Cas9* integration was found in two of the analyzed lines, namely line 26 and line 78.

**Table 1:** Overview of Genotyping Results for Genome-Edited Chicory Plants

gene of interest	tissue	number of individuals genotyped	editing efficiency (%)	genotyping approach
*CYP71DD33*	calli, 7 weeks	33	21	Sanger sequencing
	shoots, 4 months	88	22	Sanger sequencing
	regenerated lines^a^	8	100	Sanger sequencing
*CYP71DD35*	shoots, 4 months	96	36	Illumina sequencing
	regenerated lines^a^	5	100	Illumina sequencing
*CYP71DD20*	shoots, 4 months	96	10	Illumina sequencing
	regenerated lines^a^	7	100	Illumina sequencing

a Detailed description of the genotypes is given in Table S2.

The inactivation of *CYP71DD35* and *CYP71DD20* was carried out by transient transfection of chicory protoplasts with RNPs using a single guide targeting exon 1 of these genes. The genotyping of the genome-edited lines was performed at the stage of young tissue culture shoots. To this end, DNA was isolated from the leaves, and the target region was amplified by PCR and sequenced using Illumina sequencing. For *CYP71DD35,* the analysis showed 36% editing efficiency, and by this procedure, 35 genome-edited chicory lines were identified (Table 1). Five lines were fully regenerated, and the detailed genotyping of these lines revealed that two of them were heterozygous, still carrying one WT allele (Table S2). Three regenerated lines were biallelic mutants, carrying out-of-frame mutations in both alleles of *CYP71DD35*.

The genotyping of *CYP71DD20* revealed a 10% editing efficiency, and 9 genome-edited chicory lines were obtained (Table 1). From these, seven plants were fully regenerated and genotyped in detail (Table S2). One of the lines was heterozygous, still carrying one WT allele, while 6 lines showed biallelic mutations. Using a single guide for the genome editing of *CYP71DD35* and *CYP71DD20* resulted in nucleotide insertions of 1 bp or deletions, which ranged in size from 1 to 16 bp.

For guides targeting *CYP71DD33* and *CYP71DD35,* no off-target sites were predicted, while for the guide targeting *CYP71DD20,* possible off-target sites with one to two SNPs compared to the target gene were identified in the chicory genome. The genotyping analysis indeed revealed that off-target genome editing was observed for the guide targeting *CYP71DD20* in three other closely related P450 enzymes (Table S3). This could be expected as these differed only by 1–2 SNPs, and therefore, in addition to mutations in *CYP71DD20,* additional mutations in other cytochrome P450 enzymes were observed.

### Inactivation of CYP71DD33 Results in the Interruption of Lactucopicrin and Lactucin Biosynthesis and The Accumulation of 8-Deoxylactucin and Its Derivatives in Chicory Leaves

Terpene profiling of chicory leaves was performed in an early stage of plant regeneration in order to identify shoots with changes in the terpene composition. For each chicory line, a small section of the tissue culture shoot was sampled, and a single measurement was carried out to select lines for further multiplication and statistical analysis. Leaves of 14 genome-edited chicory lines showing mutations in *CYP71DD33* were sampled in tissue culture for metabolite analysis. The LC-Orbitrap-FTMS (liquid chromatography-Orbitrap-Fourier transform mass spectrometry) analysis of STLs in the leaves revealed that the accumulation of lactucin, lactucopicrin, lactucin-15-oxalate, and lactucopicrin-15-oxalate was nearly completely eliminated in 9 of the 14 chicory lines. In the same 9 chicory lines, an increased accumulation of 8-deoxylactucin, 8-deoxylactucin-15-oxalate, 11β,13-dihydro-8-deoxylactucin, and 11β,13-dihydro-8-deoxylactucin-15-oxalate was observed (Figure S4) compared to the 9 regenerated control lines. On average, a 2.5-fold increase in these compounds was observed compared to regeneration control lines. The terpene profile indicated that CYP71DD33 catalyzes the 8-hydroxylation of 8-deoxylactucin to generate lactucin in chicory. Its inactivation therefore prevents the biosynthesis of downstream STLs, lactucin, and lactucopicrin and their oxalates. Therefore, this gene was named lactucin synthase (CiLCS). Terpene profiling of leaves for 8-deoxylactucin accumulation also enabled early selection of lines for multiplication and further characterization in roots.

In contrast, the inactivation of *CYP71DD35* and *CYP71DD20* did not result in changes of the terpene profile of tissue culture leaves (Figure S4). While the overall amount of terpenes varied between the lines, production of all six major chicory STLs was still observed in all of the genome-edited chicory lines, and no new terpene products were observed. Therefore, these cytochrome P450 genes were presumed not to play a role in chicory STL biosynthesis. Nonetheless, a number of chicory lines with edits in *CYP71DD35* and *CYP71DD20* were selected for plant regeneration and terpene profiling in the chicory roots.

### Characterization of Lactucin Synthase Activity in Yeast Microsome Assays

**Figure 4:**
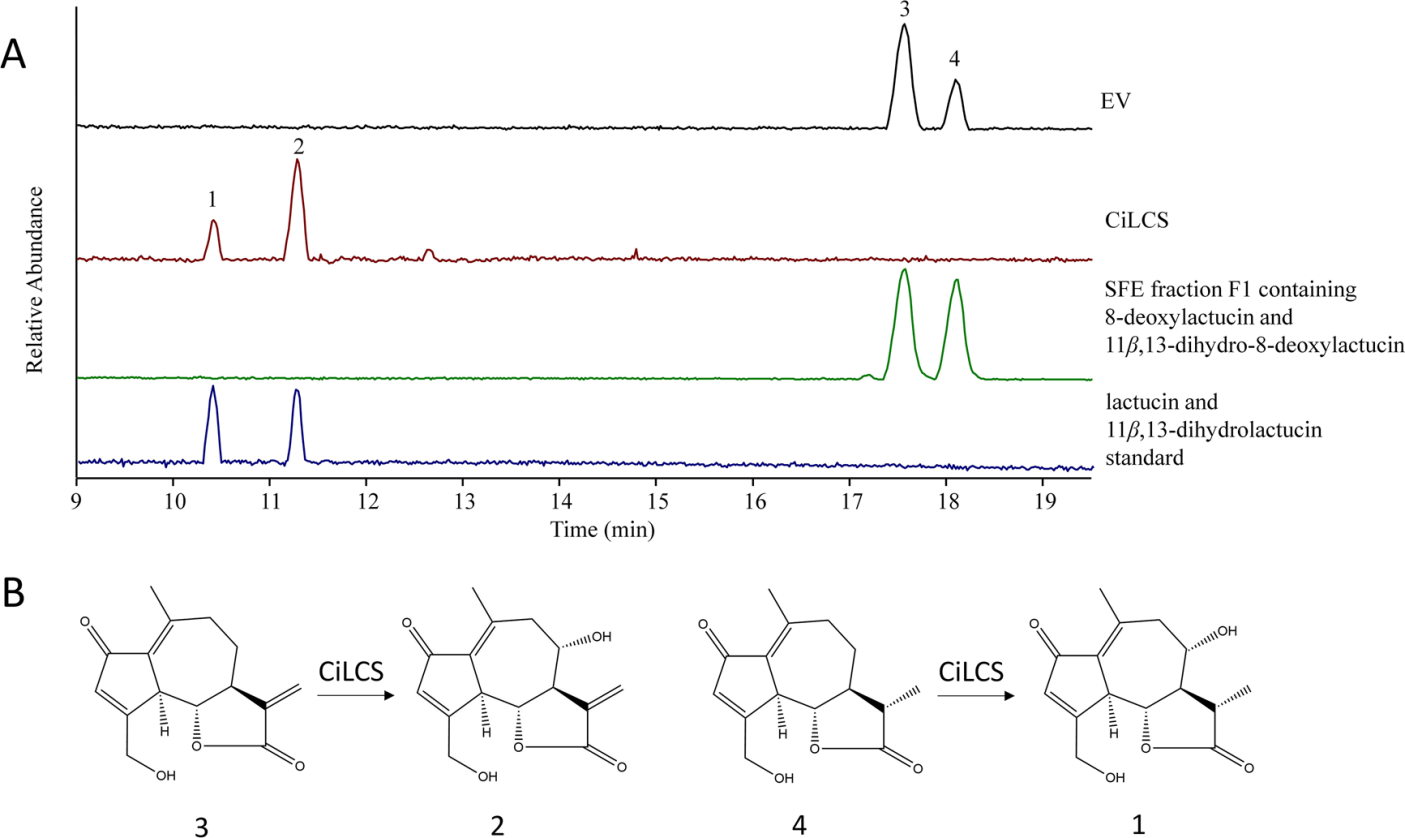
Analysis of the enzyme activity of LCS in yeast microsomes. Microsomes isolated from the WAT11 yeast strain expressing the chicory lactucin synthase gene (CiLCS, CYP71DD33) or the WAT11 strain containing the empty vector pYEDP60 (EV) were incubated with SFE fraction F1 containing 11β,13-dihydro-8-deoxylactucin and 8-deoxylactucin. Upon incubation with CiLCS, total conversion of 8-deoxylactucin and 11β,13-dihydro-8-deoxylactucin to lactucin and 11β,13-dihydrolactucin was observed. The chromatograms of the SFE fraction F1 containing 11β,13-dihydro-8-deoxylactucin and 8-deoxylactucin and a chromatogram of a mix of lactucin and 11β,13-dihydrolactucin authentic standards are shown below. Peak 1—11β,13-dihydrolactucin, [M + H]^+^ = 279.12270; peak 2—lactucin [M + H]^+^ = 277.10705, peak 3—8-deoxylactucin, [M + H]^+^ = 261.11213, peak 4—11β,13-dihydro-8-deoxylactucin, [M + H]^+^ = 263.12778. (B) Conversions catalyzed by CiLCS. Numbers correspond to peaks shown in panel A.

To confirm the putative identification of CYP71DD33 as a lactucin synthase, the corresponding gene was amplified from chicory root cDNA and expressed in yeast strain WAT11, which was constructed for the expression of animal and plant cytochrome P450 enzymes.^28^ The microsomes were isolated from a yeast strain expressing *CYP71DD33* and from a yeast strain containing the empty pYEDP60 vector. SFE extract fraction F1 containing a mixture of 8-deoxylactucin and 11β,13-dihydro-8-deoxylactucin was used as a substrate in the microsome enzyme assay. Upon incubation of the microsomes of yeast strains expressing *CYP71DD33* with fraction F1, total conversion of both substrates was observed, and the formation of two new peaks was detected by LC-Orbitrap-FTMS (Figure 4). The retention time and the accurate mass (5 ppm accuracy) of the new peaks matched with those of the authentic standards of lactucin and 11β,13-dihydrolactucin (Figure S5), indicating that the CYP71DD33 indeed catalyzes the hydroxylation of 8-deoxylactucin on the 8 position to form lactucin. This enzyme also catalyzed the hydroxylation on the 8 position of a related substrate, 11β,13-dihydro-8-deoxylactucin to form 11β,13-dihydrolactucin. This analysis confirmed that CYP71DD33 indeed has lactucin synthase activity.

### The Inactivation of CYP71DD33 Leads to the Accumulation of 8-Deoxylactucin and Its Derivatives in Chicory Taproots

Chicory taproots are the major organ for terpene production in chicory; therefore, a number of genome-edited plants were multiplied and transferred to soil in three biological replicates. Taproots were harvested after 3 months, and their terpene profile was analyzed. No visual phenotype was observed under the used growth conditions compared to the control chicory lines. The analysis of the root terpenes of genome-edited lines with mutations in *CYP71DD35* and *CYP71DD20* revealed that the profile of terpenes was not significantly changed compared to the regeneration controls (Figure S6), corresponding to the previous observation in the leaves.

**Figure 5:**
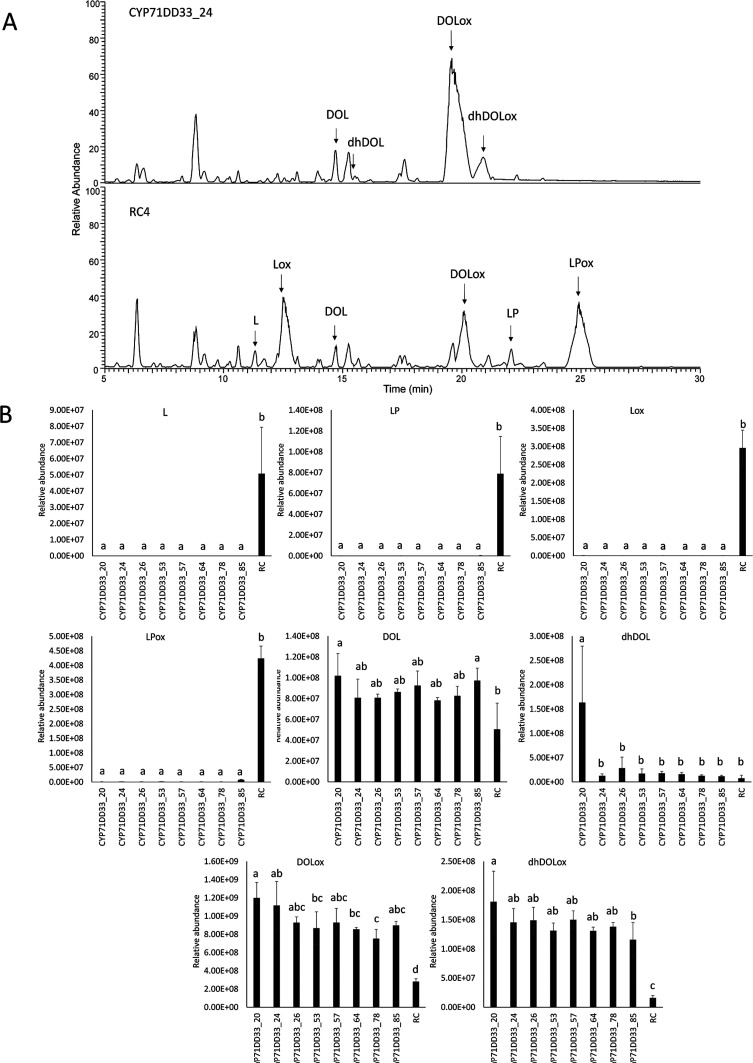
Terpene profile analysis of roots of genome-edited chicory lines CYP71DD33 by LC-Orbitrap-FTMS. (A) LC–MS chromatograms of a representative genome-edited line with a mutation in *CYP71DD33* is shown in comparison to a regeneration control line (RC). Chromatograms are shown at the same scale. *X*-axis: chromatographic retention time (min); *Y*-axis: MS detector response of the base peak in the positive ionization mode (100% corresponds to 4.0 × 10^7^ ion counts/scan). The peaks representing STLs are annotated in the chromatogram. (B) Peak areas of STLs are shown for genome-edited lines compared to the regeneration control lines. Mean and standard deviations of three biological replicates are shown for the genome-edited plants and for eight biological replicates for regeneration control lines. L—lactucin, LP—lactucopicrin, DOL—8-deoxylactucin, Lox—lactucin-15-oxalate, DOLox—8-deoxylactucin-15-oxalate, LPox—lactucopicrin-15-oxalate, dhDOL—11β,13-dihydro-8-deoxylactucin and dhDOLox—11β,13-dihydro-8-deoxylactucin-15-oxalate. The letters indicate significantly different groups according to ANOVA and the Tukey’s post hoc test (*P* < 0.05).

The analysis of taproots of chicory lines with mutations in *CYP71DD33* showed that analogous to the preliminary metabolite profiling in chicory leaves also in the taproot lactucin, lactucopicrin and the corresponding oxalates were eliminated (Figure 5). In the 8 analyzed lines, the presence of 8-deoxylactucin and 11β,13-dihydro-8-deoxylactucin was detected. In comparison to the regeneration control lines, the most significant increase was observed in the accumulation of oxalated 8-deoxylactucin derivatives 8-deoxylactucin-15-oxalate and 11β,13-dihydro-8-deoxylactucin-15-oxalate. The content of the dihydro-STLs was on average sixfold lower compared to 8-deoxylactucin and 8-deoxylactucin-15-oxalate. In the 8 analyzed lines, the total content of 8-deoxylactucin and its derivatives was increased from 2.7- to 4.5-fold. When comparing the total peak area of all terpenes, no significant difference was found between genome-edited chicory lines and regeneration control lines, indicating that the total amount of terpenes was not changed. The content of major chicory taproot phenolic compounds, chlorogenic acid, and isochlorogenic acid^29^ did not significantly differ between regeneration control lines and chicory lines with mutations in *CYP71DD33*. In conclusion, chicory roots were obtained that accumulated 8-deoxylactucin and its derivatives as the major STLs.

## Discussion

In this study, we confirmed the anti-inflammatory potential of a fraction of chicory STLs enriched in 8-deoxylactucin and 11β,13-dihydro-8-deoxylactucin, as demonstrated previously in a yeast reporter system based on the activity of Crz-1, the yeast orthologue of the human NFAT (nuclear factor of activated T-cells).^9^ In the present study, we showed that an 8-deoxylactucin and 11β,13-dihydro-8-deoxylactucin-rich chicory extract fraction (F1) has anti-inflammatory activity in a physiologically relevant model of the inflamed intestinal mucosa composed of three cell types that can be found in the human intestinal epithelium: absorptive enterocytes (Caco-2), mucus-secreting goblet cells (HT29-MTX), and antigen-uptake facilitator microfold (M)-cells (RajiB-induced). In this triple co-culture model, which comprises the complex and representative features of a biological environment characteristic of inflammatory bowel disease (IBD), fraction F1 was able to decrease the release of IL-8 and the protein expression levels of iNOS. Such results are an important step forward in elucidating biological mechanisms of these compounds relevant for IBD and in evaluating the anti-inflammatory potential of uncharacterized STLs from chicory for the treatment of inflammatory conditions.

IL-8 is a relevant pro-inflammatory chemokine secreted by several cell types and tissues with the main goal of recruiting immune cells, particularly neutrophils, from the blood stream, thus being commonly used as a biomarker of inflammation.^30^ The systemic-target nature of IL-8 may justify the much higher concentrations found in the basolateral rather than in the apical compartment of the cell model since the former represents the systemic circulation and the latter depicts the intestinal lumen.

Unlike the constitutive isoforms of NOS, eNOS, and nNOS, iNOS is an inducible isoform of the enzyme that is expressed in several tissues upon an inflammatory stimulus, resulting in increased production of reactive oxygen species, vascular permeability, and edema due to the high levels of nitric oxide (NO).^31^ Increased protein expression of iNOS is a result of the activation of numerous signal transduction cascades, including the NF-κB pathway.^32^ For this reason, BMS 345541, an IKK-1/IKK-2 inhibitor, was used as a positive control for the inhibition of the NF-κB pathway and therefore the expression of iNOS. The ability of F1 to decrease iNOS expression to levels close to those obtained for BMS 345541 suggests that the compounds present in this fraction, namely 8-deoxylactucin and 11β,13-dihydro-8-deoxylactucin, may be able to hamper NF-κB pathway activation in a similar extent to that of the positive control. These results confirm the promising anti-inflammatory effect of F1 and therefore 8-deoxylactucin and 11β,13-dihydro-8-deoxylactucin. However, further studies need to be conducted to unravel the mechanisms of action by which the STLs present in F1 exert their anti-inflammatory effect. Since these compounds are not commercially available, we cannot test them individually to determine whether 8-deoxylactucin or its derivative 11β,13-dihydro-8-deoxylactucin has a more effective anti-inflammatory potential. Nonetheless, it is worthy of note that, as opposed to the α-methyl-γ-lactone found in 11β,13-dihydro-8-deoxylactucin, 8-deoxylactucin has an α-methylene-γ-lactone moiety, which is the main structural feature responsible for STL reactivity.^7^ However, the presence of such a structure may not be straightforwardly related to an improved bioactivity, as was previously observed for 11β,13-dihydrolactucin vs lactucin in a yeast reporter system based on the activity of Crz1.^8^

Considering these bioactivity results, we set out to generate chicory lines that preferentially accumulate 8-deoxylactucin and its derivatives to high levels in chicory roots. For this purpose, the enzyme that catalyses the conversion of 8-deoxylactucin to lactucin needs to be inactivated. Three cytochrome P450 candidate genes were identified, and gene inactivation *in planta* and subsequent confirmation of activity by yeast expression experiments confirmed CYP71DD33 to be responsible for this conversion in chicory. This enzyme was designated as chicory lactucin synthase (CiLCS). *Cichorium endivia* and *Lactuca* species are also known to produce lactucin^3^ and are expected to express lactucin synthases. Blast analysis of the NCBI nucleotide collection (nr/nt) revealed that the closest full-length homologue of CYP71DD33 is found in *L. sativa* with 78% amino acid identity. When the NCBI-expressed sequence tag (est) database was queried, partial-length hits with >95% amino acid identity were found in *C. endivia* and homologues with >75% amino acid identity were found in several *Lactuca* species, namely *L. sativa*, *L. virosa*, *Lactuca saligna,* and *Lactuca serriola*. It is not yet known if the genes in these plants also encode lactucin synthases. Thus, CYP71DD33 from chicory is the first lactucin synthase enzyme identified in plants.

In chicory, the network of the laticifers is tightly connected to the phloem in the vascular bundles, and it contains high amounts of bitter compounds. Recently, it was shown that the gene of the first dedicated step of STL biosynthesis catalyzed by the germacrene A synthase is expressed in vascular parenchyma cells neighboring laticifers in lettuce.^33^ Our study reveals that *CiLCS* is highly expressed in the chicory latex as was also observed for the chicory kauniolide synthase *CiKLS* gene.^14^ These findings indicate that the late steps of the STL biosynthesis in chicory are tissue-specific and localized in the latex.

Genome editing has been used successfully in chicory to alter the terpene composition of the taproots. Previously, the interruption of the *CiGAS* gene was shown to lead to chicory roots that lack STLs.^23^ This may benefit the extraction of inulin from chicory roots, as bitter STLs need to be removed during processing. When a later biosynthetic step catalyzed by the chicory kauniolide synthase CiKLS, leading from costunolide to kauniolide, was inactivated by CRISPR/Cas9, chicory taproots were found to accumulate costunolide and its conjugates.^14^ In the current study, three different P450 genes were inactivated, resulting in the case of CYP71DD33 in strong enrichment of 8-deoxylactucin and its derivatives in taproots. The plants showed no visual phenotypic difference compared to the control chicory lines. Additionally, while conjugation of costunolide to cysteine and glutathione was observed for costunolide-accumulating chicory lines, the 8-deoxylactucin was not found to be conjugated to cysteine or glutathione in these genome-edited plants, nor is it in wild-type plants. The conjugation may serve as a detoxification strategy in the case of costunolide, which is, in contrast to 8-deoxylactucin, an intermediate that does not accumulate in wild-type chicory plants. However, any putative toxicity of 8-deoxylactucin to chicory root cells may be prevented by its conjugation to oxalate which is predominant in chicory.^3^

Genome editing by plasmid and RNP approaches apparently works very efficiently in chicory, since in the case of *CiGAS* and *CiKLS*, eight and six alleles, respectively, were successfully inactivated, and in this study, three more cytochrome P450 genes were interrupted. This shows the potential of metabolic engineering using genome editing in chicory to generate new varieties and control the production of STLs with interesting bioactivity in its taproots. Costunolide and its conjugates,^14^ which have been documented to have anti-cancer activity, and the anti-inflammatory 8-deoxylactucin and derivatives (this study) accumulated in the CiKLS and CiLCS loss-of-function chicory lines to high levels, similar to the amounts of total STLs in wild type chicory. With the recently developed supercritical CO_2_ extraction protocol^9^ which can be applied at an industrial scale and is compatible with inulin extraction, chicory can be positioned as a multipurpose crop from which different health products can be extracted.

## Materials and Methods

### Caco-2 Cell Culture

Human colon carcinoma Caco-2 cells (DSMZ, Braunschweig, Germany), mucus-secreting HT29-MTX-E12 subclone (ECACC, Dublin, Ireland), and human Burkitt’s lymphoma Raji B cells (ECACC, Oxford, UK) were routinely grown separately in high glucose, high pyruvate, Dulbecco’s modified Eagle medium (DMEM) (Gibco, Life Technologies, Grand Island, NY, USA), supplemented with 10% (v/v) of heat-inactivated fetal bovine serum (FBS) (Biowest, Nuaillé, France), 100 units/mL penicillin, 100 μg/mL streptomycin (Gibco, Life Technologies, Grand Island, NY, USA), and 10 mM nonessential amino acids (Gibco, Life Technologies, Paisley, UK). Cells were cultured in a humidified atmosphere at 37 °C with 5% CO_2_.

### Caco-2/HT29-MTX-E12/RajiB Cell Co-culture

According to Lozoya-Agullo et al.,^34^ Caco-2 and HT29-MTX-E12 cells were co-cultured at a proportion of 9:1, respectively, on the apical chamber of 12 mm Transwell inserts (polyester membrane, 0.4 μm pore size, Corning CoStar Corp., NY, USA) at a total density of 1.0 × 10^5^ cell/cm^2^. After 14 days of the double co-culture growth, RajiB cells were added to the basolateral compartment at a density of 4.0 × 10^4^ cell/mL, and the triple co-culture was maintained further for another 7 days. The culture medium was changed three times per week for cell culture maintenance purposes. The triple co-culture-inflamed intestinal mucosa model was based on previous studies using only Caco-2 cells^35^ and was validated by several methodologies, namely the LDH release assay (Biolegend, CA, USA) and resazurin metabolization (PrestoBlue, Invitrogen, Thermo Fisher Scientific, MA, USA) for cell viability, transepithelial electrical resistance (TEER) monitorization, sodium fluorescein permeability, and IL-8 release (Figure S1).

To ensure monolayer formation and integrity, TEER was monitored throughout the culture period using an EVOM voltmeter (WPI, Berlin, Germany).

### Inflammation Assays

Before the inflammation assays, cells were washed with DPBS (Corning, VA, USA) and incubated with the samples of interest [chicory SFE or 8-deoxylactucin/11β,13-dihydro-8-deoxylactucin-enriched SFE fraction 1 (F1)] in the presence of a pro-inflammatory stimulus consisting of 10 μg/mL LPS from *E. coli* (Sigma-Aldrich, MO, USA) on the apical chamber and 25 ng/mL IL-1β (Sino Biological, Eschborn, Germany) and 50 ng/mL TNF-α (Peprotech, NJ, USA) on the basolateral chamber. SFE or F1 were added to the apical compartment. Besides the SFE, F1 was selected after being tested along with two other fractions isolated from SFE (Figure S2).

Before each experiment, TEER was measured before, and only monolayers with a value higher than 400 Ω·cm^2^ after 21 days were used for inflammation assays.

For ELISA measurements of IL-8 release, the inflammation assay as described above was carried out for 48 h; for iNOS protein expression assessment by Western blot, the inflammatory stimulus was sustained for 12 h before cell pellet collection.

### Determination of IL-8 Levels

After a 48 h co-incubation of cells with the sample of interest and an inflammatory stimulus, apical and basolateral supernatants were collected, snap-frozen with liquid nitrogen, and stored at −80 °C until the day of analysis. IL-8 levels were measured by ELISA following manufacturer’s instructions (product Nr. 900-T18, Peprotech, NJ, USA). Briefly, the sandwich ELISA method was used, consisting of an overnight incubation with the capture antibody prior to the addition of the IL-8-containing samples, followed by an incubation with the detection antibody, and finally the addition of a streptavidin-horseradish peroxidase conjugate. The results were obtained by absorbance measurements at 450 and 620 nm after development with the 3,3′,5,5′-tetramethylbenzidine liquid substrate and addition of 1 M hydrochloric acid as a stop solution. Each supernatant sample was diluted with phosphate buffered saline to fit the IL-8 calibration curve (0–200 pg/mL) and tested in at least two technical replicates per sample.

### Assessment of iNOS Expression

Following a 12 h pro-inflammatory co-incubation of the co-culture with SFE, F1, or the IKK-1/IKK-2 inhibitor BMS 345541 (Abcam, Cambridge, UK) (used as a positive control), cells were washed twice with ice-cold PBS, cell monolayers were scraped from the Transwell insert membranes, and technical replicates were pooled. After a 5 min centrifugation at 5000*g*, the supernatant was discarded, and cell pellets were snap-frozen and stored at −80 °C until further analysis. Total protein extraction was carried out with RIPA lysis and extraction buffer (Thermo Scientific, IL, USA), and protein quantification was performed with the Micro BCA Protein Assay Kit (Thermo Scientific, IL, USA), following the manufacturer’s instructions. 40 micrograms of protein of each sample were separated in SDS-PAGE gels at 120 V and transferred into nitrocellulose membranes (Bio-Rad, Feldkirchen, Germany) at 0.37 mA. After blocking with 5% BSA in TBST (tris buffered saline solution containing 0.05% (v/v) Tween-20) for 1 h at room temperature, the membranes were incubated overnight at 4 °C with either rabbit anti-iNOS (1:000) or mouse anti-β-actin (1:1000) primary antibodies (Cell Signaling Technology, MA, USA) in TBST containing 2.5% BSA. Incubation with species-specific HRP-conjugated secondary antibodies was performed for 1 h at room temperature using sheep anti-rabbit (1:2000) and horse anti-mouse (1:3000) antibodies (Cell Signaling Technology, MA, USA). The signal was then detected using the Clarity Western-enhanced chemiluminescence (ECL) detection kit (Bio-Rad, CA, USA) in an iBright FL1500 transilluminator (Invitrogen, Thermo Fisher Scientific, MA, USA).

### Gene Isolation and Phylogenetic Analysis

Three candidate cytochrome P450 genes were identified in the chicory genome (NCBI BioProject PRJNA901408) by sequence homology to HaES and TpPTS. The encoded proteins were classified into the CYP71 class of cytochrome P450 enzymes and were assigned the names CYP71DD20, CYP71DD33, and CYP71DD35 by Dr. David Nelson.^26^ The cDNA and protein sequence were submitted to NCBI under accession numbers OP973198, OP973199, and OP973200. Multiple protein sequence alignments were performed using CLC genomics workbench 20.0.4 software (Qiagen). An unrooted phylogenetic tree was constructed to compare the protein sequences of CYP71DD20, CYP71DD33, and CYP71DD35 to other cytochrome P450 enzymes described to catalyze the hydroxylation of germacrene A, germacrene A acid, or costunolide, namely *C. intybus* germacrene A oxidase (CiGAO),^11^*L. sativa* germacrene A oxidase (LsGAO),^11^*C. intybus* costunolide synthase (CiCOS),^12^*L. sativa* costunolide synthase (LsCOS),^13^*Helianthus annuus* germacrene A acid 8β-hydroxylase (HaG8H),^17^*Inula hupehensis* germacrene A acid 8β-hydroxylase (IhG8H),^18^*C. intybus* kauniolide synthase 1 (CiKLS1),^14^*Tanacetum parthenium* kauniolide synthase (TpKLS),^15^*H. annuus* costunolide 14-hydroxylase (HaC14H),^16^*T. parthenium* 3-beta hydroxylase (Tp3BH),^21^*T. parthenium* parthenolide synthase (TpPTS),^21^ and *H. annuus* eupatolide synthase (HaES).^17^ Bootstrap N-J trees were generated with 100 replicates of bootstrap analysis in CLC genomics workbench 20.0.4 software (Qiagen).

### Gene Expression Analysis

Gene expression of P450s was studied in a dataset of Illumina pair-based read data of different chicory taproot tissues including cortex, hypodermis, phloem, vascular cylinder, laticifer, and latex tissues sampled in triplicate (NCBI BioProject PRJNA824299). Illumina reads were mapped to the genomic sequences of P450s using CLC genomics workbench 20.0.4 (Qiagen). The paired reads were each counted as one mapped read. The normalized gene expression per each tissue was calculated as reads per kilo base per million mapped reads (RPKM). Data were subjected to statistical analysis using SPSS software (version 28 for Windows; IBM, Armonk, NY, USA) for the analysis of variance (ANOVA). Tukey’s post hoc test (*P* > 0.05) was used to analyze the differences between tissues.

### CRISPR/Cas Inactivation of *CYP71DD33*, *CYP71DD35,* and *CYP71DD20*

For the inactivation of CYP71DD33, four guide RNAs (sgRNAs) targeting exon 1 of the corresponding gene were designed (Table S1). The target sites of the four guide RNAs were located close to each other within a 120 bp region to enable efficient genotyping. The four guides, each of them in combination with the *Arabidopsis thaliana* U6 promoter, were synthesized as a single synthetic DNA fragment (Genscript) and cloned into the pUC57 plasmid (Genscript). By this procedure, plasmid pUC57-pU6::sgRNA9-pU6::sgRNA13-pU6::sgRNA21-pU6::sgRNA23 was generated which was directly used for chicory protoplast transfection. The MoClo toolkit and Golden Gate assembly were used to assemble the vector for SpCas9 expression.^36^ To this end, the *A. thaliana* codon-optimized *SpCas9* ORF, the constitutive parsley ubiquitin promoter, and the NOS terminator were cloned into a Level 1 vector backbone plCH47742, generating plasmid plCH47742-PcUBp::aCas9::tNOS. This plasmid was used directly for protoplast transfection. *C. intybus* subsp. *intybus* var. *sativum* cultivar Orchies was used in the study. The *in vitro* clone C37 was selected due to high transformation and regeneration capacity. The transfection of protoplasts with plasmids, regeneration of protoplasts, and plant cultivation were done as previously described.^14^ For the RNP approach, the Cas9 protein (EnGen Spy-NLS) was obtained from New England Biolabs, and the guides were produced using the EnGen sgRNA synthesis kit (E3322V) following the manufacturer’s instructions. The transfection of the CRISPR/Cas9 reagents, regeneration of plants, and plant cultivation were done according to the protocol described in Cankar et al.^23^

### Genotyping Chicory Plants

The genotyping of calli and shoots of chicory lines with mutations in *CYP71DD33* was performed by Sanger sequencing. A PCR product was amplified from a small sample of the leaf tissue using the Phire Plant Direct PCR Kit (Thermo Fisher) using primers targeting *CYP71DD33*. The PCR products were sent for Sanger sequencing and analyzed for the presence of indels at the target sites. For detailed genotyping of eight mutant lines showing large differences in the terpene profile, PCR products were subsequently purified and cloned into the pJET1.2 vector (Thermo Fisher), and several cloned PCR products were sequenced by Sanger sequencing. Sequence data were analyzed by SeqMan Pro software (DNASTAR). *Cas9* gene integration was checked by PCR using specific primers that amplified an 800 bp fragment of the *Cas9* gene.

To assess the efficiency of genome editing for lines with mutations in *CYP71DD35* and *CYP71DD20,* a PCR product was amplified from a small sample of the leaf tissue using specific primers (Table S1). A nested PCR was done on each PCR product, and a final third PCR was performed with barcoded Illumina primers to enable later identification of the sequences. All of these PCR products were then pooled and paired-end sequenced on an Illumina MiSeq apparatus. The sequences were analyzed for the presence of indel mutations at the target sites.

### Cloning of *CYP71DD33* from Chicory for Expression in Yeast

The coding sequence (CDS) of *CYP71DD33* was amplified by PCR from chicory taproot cDNA while at the same time introducing *Not*I and *Pac*I restriction sites.^37^ The CDS was subsequently cloned into a yeast expression vector pYEDP60, which was modified to contain *Not*I and *Pac*I restriction sites at the polylinker.^28,38^ pYEDP60 plasmids containing *CYP71DD33* and the empty vector pYEDP60 were transformed into the yeast strain WAT11^39^ using standard procedures.^40^ The recombinant yeast colonies were selected on solid synthetic dextrose minimal medium (SD medium: 0.67% Difco yeast nitrogen base medium without amino acids, 2% d-glucose, 2% agar) supplemented with amino acids, but omitting uracil and adenine sulfate for auxotrophic selection.

### Microsome Preparation and the *In Vitro* Enzyme Assay

Microsomes from yeast cultures were prepared as previously published.^15,37^ Microsomal preparation from WAT11 cultures containing an empty pYEDP60 plasmid was used as a negative control. Enzyme assays were conducted in a total volume of 500 μL, containing 40 mM KPi buffer (pH = 7.5), 2% DMSO, 2 mM NADPH, and 100 μL of microsomal preparation. The supercritical CO_2_ extract of chicory root STLs was isolated and fractionated as described previously.^9^ Fraction F1 of the extract contained 8-deoxylactucin and 11β,13-dihydro-8-deoxylactucin. This fraction was dissolved in DMSO at the concentration of 10 mg/mL, and 10 μL was added to the enzyme assay mixture. The enzymatic reactions were incubated for 2 h at 25 °C at 250 rpm. The reactions were stopped by the addition of an equal volume of methanol containing 0.1% formic acid; the samples were then vortexed, sonicated for 15 min, and centrifuged at 21,000*g* at room temperature. The clear supernatant was transferred to a fresh vial and used for LC-Orbitrap-FTMS analysis. LC-Orbitrap-FTMS analysis was performed as described below.

### Analysis of Sesquiterpene Lactones by LC–MS

Chicory leaf and root material were frozen and powdered in liquid nitrogen. Extraction from 300 mg of tissue was performed using methanol containing 0.1% formic acid; the samples were then vortexed, sonicated for 15 min and centrifuged at 21,000*g* at room temperature. The clear supernatant was transferred to a fresh vial and used for LC–MS analysis. LC–MS analysis was performed using a LC-PDA-LTQ-Orbitrap FTMS system (Thermo Scientific), which consists of an Acquity UPLC (H-Class) with an Acquity eLambda photodiode array detector (220–600 nm) connected to a LTQ/Orbitrap XL hybrid mass spectrometer equipped with an electrospray ionization (ESI) detector. The injection volume was 5 μL. Chromatographic separation was performed on a reversed phase column (Luna C18/2,3 μ, 2.0 × 150 mm; Phenomenex, USA) at 40 °C. Degassed eluent A [ultra-pure water: formic acid (1000:1, v/v)] and eluent B [acetonitrile/formic acid (1000:1, v/v)] were used at a flow rate of 0.19 mL/min. A linear gradient from 5 to 75% acetonitrile (v/v) in 45 min was applied, which was followed by 15 min of washing and equilibration. FTMS full scans (*m*/*z* 90.00–1350.00) were recorded with a resolution of 60,000 fwhm. Data were subjected to statistical analysis using SPSS software (version 28 for Windows; IBM, Armonk, NY, USA) for the analysis of variance (ANOVA). Tukey’s post hoc test (*P* > 0.05) was used to analyze differences between lines.

## Supporting Information Available

Pro-inflammatory stimulus produces a significant inflammatory response in a triple co-culture composed of Caco-2/HT29-MTX/RajiB cells, rationale for the choice of SFE fraction F1 over fractions F2 and F3, protein sequence analysis of candidate cytochrome P450 enzymes, terpene profiling of tissue culture leaves of genome-edited chicory lines by LC-Orbitrap-FTMS, mass spectra of lactucin and 11β,13-dihydrolactucin produced in microsomal assays, terpene profiling of taproots of genome-edited chicory lines with mutations in *CYP71DD35* and *CYP71DD20* by LC-Orbitrap-FTMS, primers and guide RNAs used in this study, detailed genotyping of mutations in regenerated genome-edited chicory lines, and off-target genome editing by guide targeting *CYP71DD20* (PDF)
