# Supplementary material for: Lactucin Synthase Inactivation Boosts the Accumulation of Anti-inflammatory 8-Deoxylactucin and Its Derivatives in Chicory (Cichorium intybus L.)
Source: J Agric Food Chem. 2023 Apr 10;71(15):6061–72. doi: 10.1021/acs.jafc.2c08959 (PMC10119987; doi:10.1021/acs.jafc.2c08959)
Supplement: Supplementary file 1 — jf2c08959_si_001.pdf [file jf2c08959_si_001.pdf]

Figure S1: Pro-inflammatory stimulus produces a significant inflammatory response in a triple co-culture composed of Caco-2:HT29-MTX:RajiB cells. A) Triple co-culture cell viability after a 48 h incubation with the pro-inflammatory stimulus assessed by LDH release and resazurin metabolic reduction. B) Percentage of TEER change after 48 h in the absence and presence of the inflammatory stimulus. C) Percentage of fluorescein apparent permeability and clearance after a 48 h inflammatory stimulation compared to the unstimulated control. D) Percentage of IL-8 release assessed by ELISA in both apical and basolateral supernatants after a 48 h inflammatory stimulus compared to the unstimulated control. All results were obtained from at least three independent biological replicates. *p<0.05, ***p<0.001.

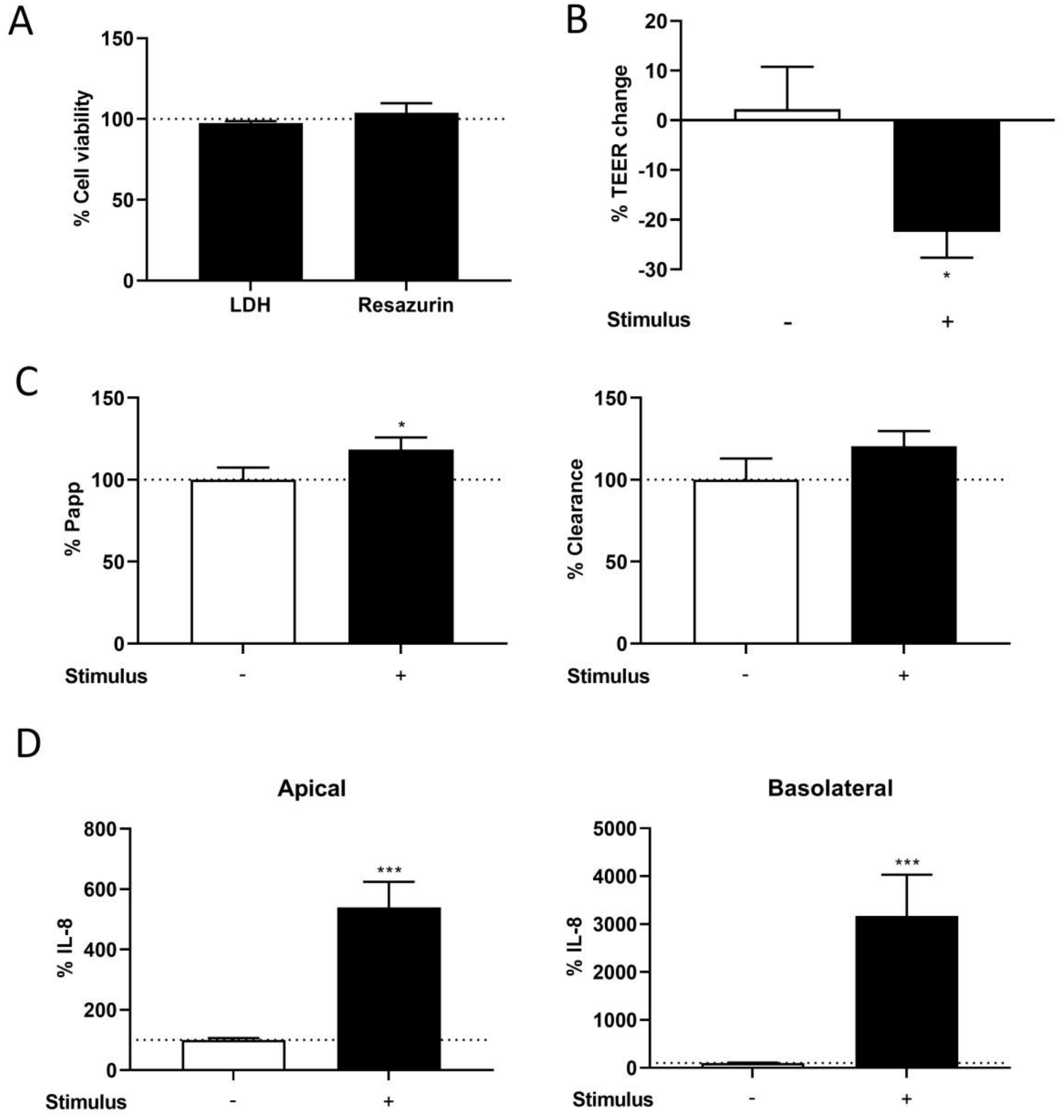

Figure S2: Rationale for the choice of SFE fraction F1 over fractions F2 and F3. A) Cytotoxicity testing of SFE and the three obtained fractions assessed by resazurin metabolic reduction in Caco-2. B) Sesquiterpene lactone composition of the highest non-cytotoxic concentrations of SFE and respective fractions. C) Percentage of IL-8 release assessed by ELISA in both apical and basolateral supernatants of cells treated with either SFE or one of the three SFE fractions for 48 h in co-incubation with the pro-inflammatory stimulus, when compared to the untreated stimulated control. D.) IL-8 release assessed by ELISA in both apical and basolateral supernatants of cells treated with F2, F3, or a mix of the main compounds present therein, for 48h in co-incubation with the pro-inflammatory stimulus. All results were obtained from at least three independent biological replicates. *$p<0.05$, **$p<0.01$, ***$p<0.001$ relative to the stimulated control.

A

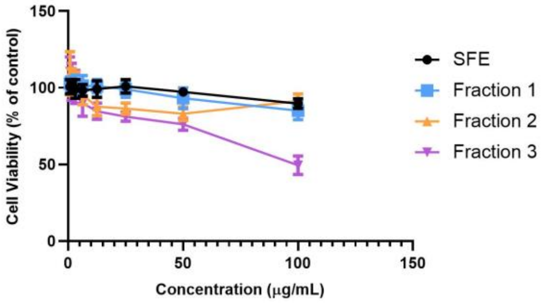

Maximum non-cytotoxic concentration	
SFE	50 μg/mL
Fraction 1	50 μg/mL
Fraction 2	5 μg/mL
Fraction 3	5 μg/mL

B

	μM SL					
**Fraction**	Lactucin	11β,13-dihydrolactucin	Lactucopicrin	11β,13-dihydrolactucopicrin	8-deoxylactucin	11β,13-dihydro-8-deoxylactucin
**50 μg/mL SFE**	**2.30**	**4.85**	**1.45**	**0.25**	1.20 μM lactucin equivalents	
**50 μg/mL Fraction 1**	Not detected	Not detected	Not detected	**0.50**	4.5 μM lactucin equivalents	
**5 μg/mL Fraction 2**	Not detected	Not detected	**0.52**	**0.07**	Not detected	Not detected
**5 μg/mL Fraction 3**	**7.49**	**8.94**	Not detected	Not detected	Not detected	Not detected

C

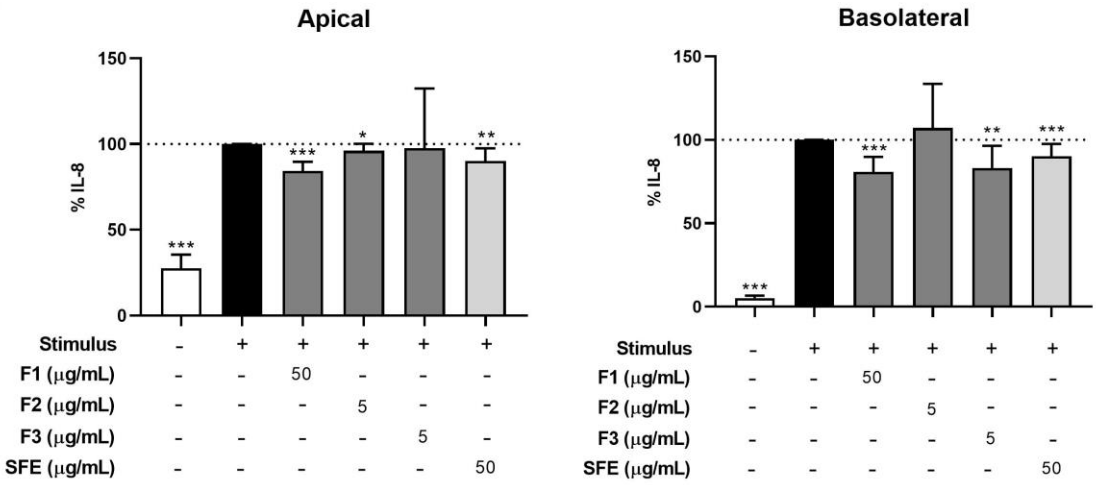

D

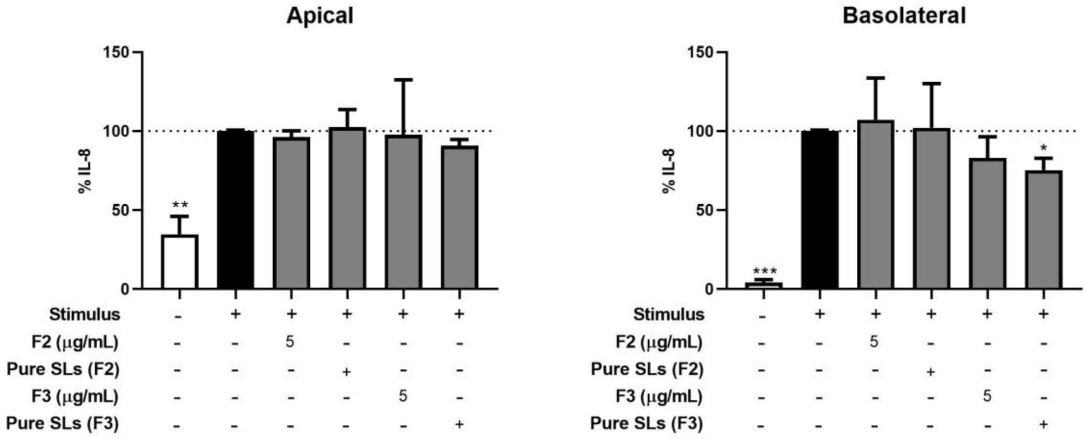

Figure S3: Protein sequence analysis of candidate cytochrome P450 enzymes. A.) The unrooted phylogenetic tree of chicory P450 enzyme candidates CYP71DD33, CYP71DD35 and CYP71DD20 and cytochrome P450 enzymes that were previously described to be involved in STL biosynthesis in the Asteraceae family, namely *Cichorium intybus* germacrene A oxidase (CiGAO), *Lactuca sativa* germacrene A oxidase (LsGAO), *C. intybus* costunolide synthase (CiCOS), *L. sativa* costunolide synthase (LsCOS), *Helianthus annuus* germacrene A acid 8β-hydroxylase (HaG8H), *Inula hupehensis* germacrene A acid 8β-hydroxylase (IhG8H), *C. intybus* kauniolide synthase (CiKLS1), *Tanacetum parthenium* kauniolide synthase (TpKLS), *T. parthenium* 3β-hydroxylase (Tp3BH), *Helianthus annuus* costunolide 14-hydroxylase (HaC14H), *T. parthenium* parthenolide synthase (TpPTS), and *Helianthus annuus* eupatolide synthase (HaES). B.) Protein alignment of chicory cytochrome P450 enzymes CYP71DD33, CYP71DD35 and CYP71DD20 and the TpPTS and HaES is shown. C. Protein sequence identity matrix.

A

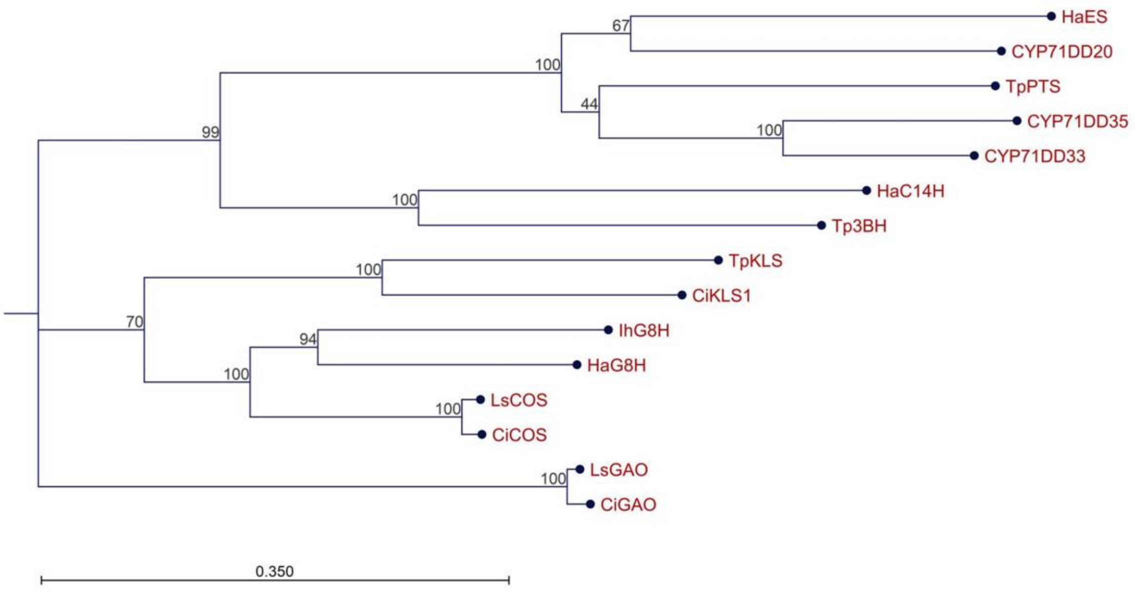

B

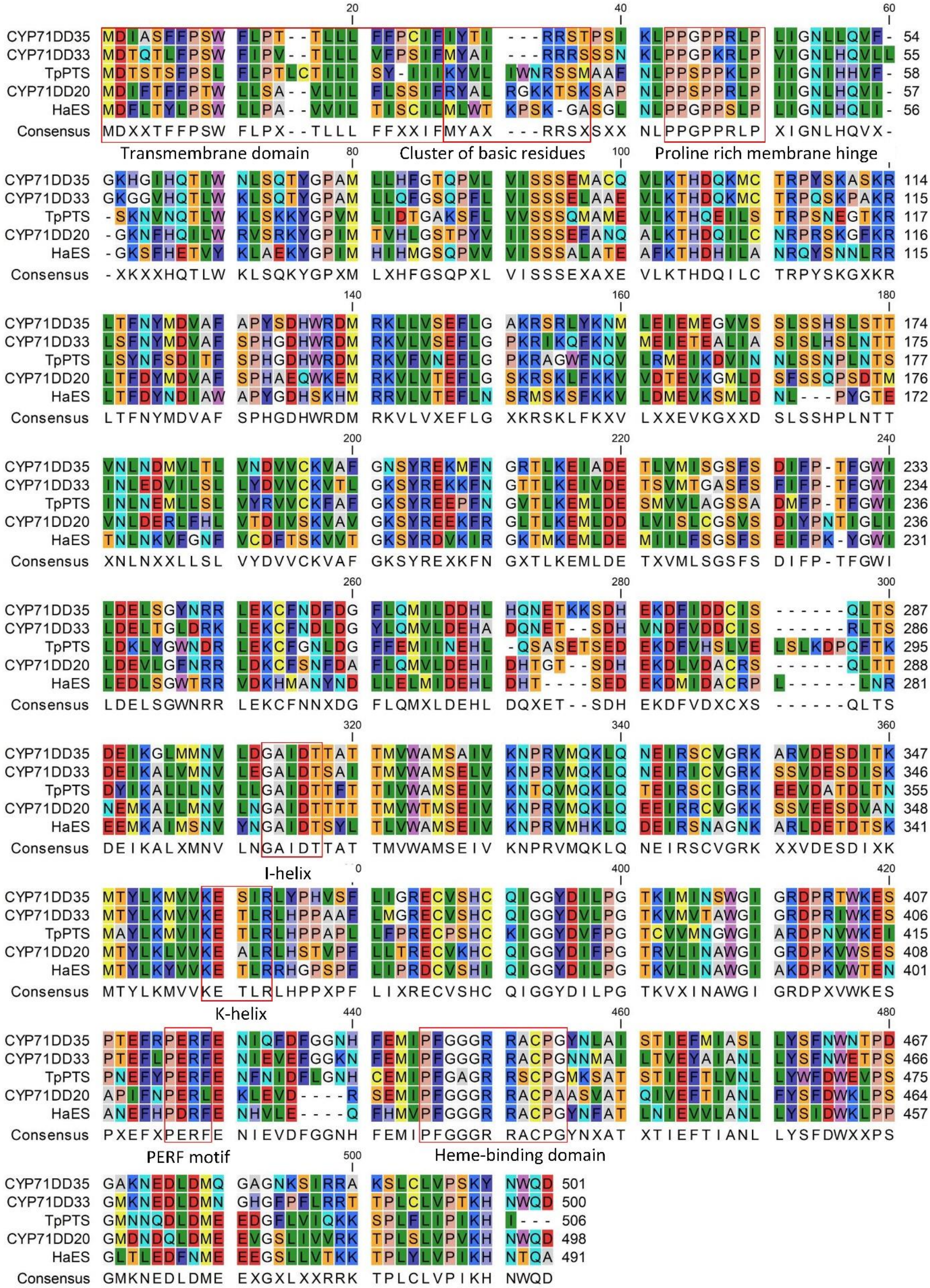
CYP71DD35
CYP71DD33
TpPTS
CYP71DD20
HaES
Consensus
Transmembrane domain
Cluster of basic residues
Proline rich membrane hinge
I-helix
K-helix
PERF motif
Heme-binding domain

C

*Percent identity \ **Identities	CYP71DD35	CYP71DD33	TpPTS	CYP71DD20	HaES
CYP71DD35		364	275	286	243
CYP71DD33	72.5		277	287	242
TpPTS	53.7	54.0		258	230
CYP71DD20	56.6	56.9	50.4		274
HaES	48.0	47.9	44.9	54.9	

*The percentage of overlapping alignment position where the two sequences agree

**The number of overlapping alignment position where the two sequences agree

Figure S4: Terpene profiling of tissue culture leaves of genome edited chicory lines by LC-Orbitrap-FTMS. For each chicory line a small section of the tissue culture shoot was sampled at an early stage and a single measurement was carried out to select lines for further multiplication. Peak areas of main sesquiterpene lactones are shown for A) Genome edited chicory lines of gene *CYP71DD33*, B) Genome edited chicory lines of gene *CYP71DD35*, B) Genome edited chicory lines of gene *CYP71DD20*. Genome edited lines are compared to regeneration control lines (RC). L - lactucin, LP - lactucopicrin, DOL - 8-deoxylactucin, Lox - lactucin-15-oxalate, DOLox - 8-deoxylactucin-15-oxalate, LPox - lactucopicrin-15-oxalate, dhDOL - 11*β*,13-dihydro-8-deoxylactucin, dhDOLox - 11*β*,13-dihydro-8-deoxylactucin-15-oxalate.

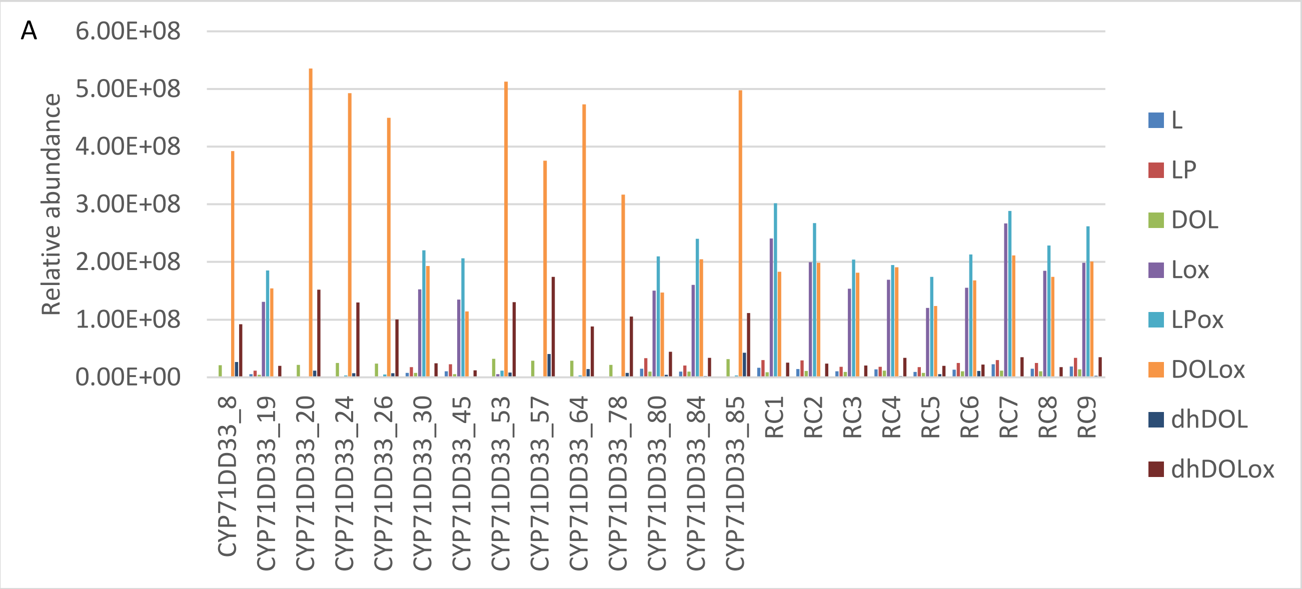

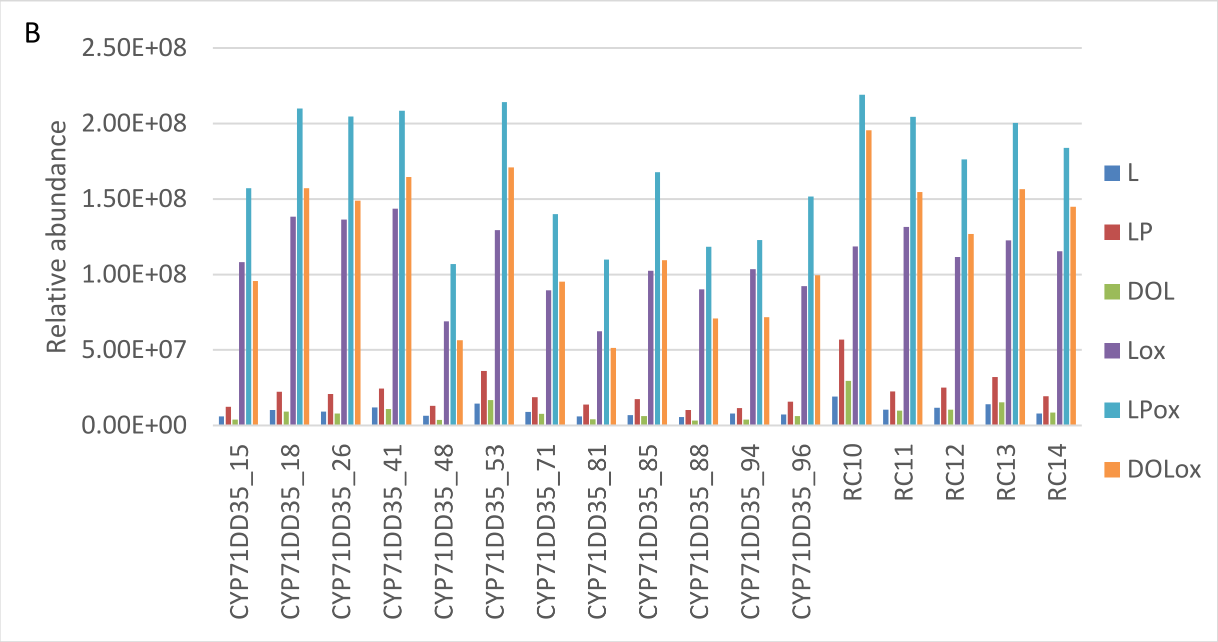
B
Relative abundance
2.50E+08
2.00E+08
1.50E+08
1.00E+08
5.00E+07
0.00E+00
CYP71DD35_15
CYP71DD35_18
CYP71DD35_26
CYP71DD35_41
CYP71DD35_48
CYP71DD35_53
CYP71DD35_71
CYP71DD35_81
CYP71DD35_85
CYP71DD35_88
CYP71DD35_94
CYP71DD35_96
RC10
RC11
RC12
RC13
RC14
L
LP
DOL
Lox
LPox
DOLox

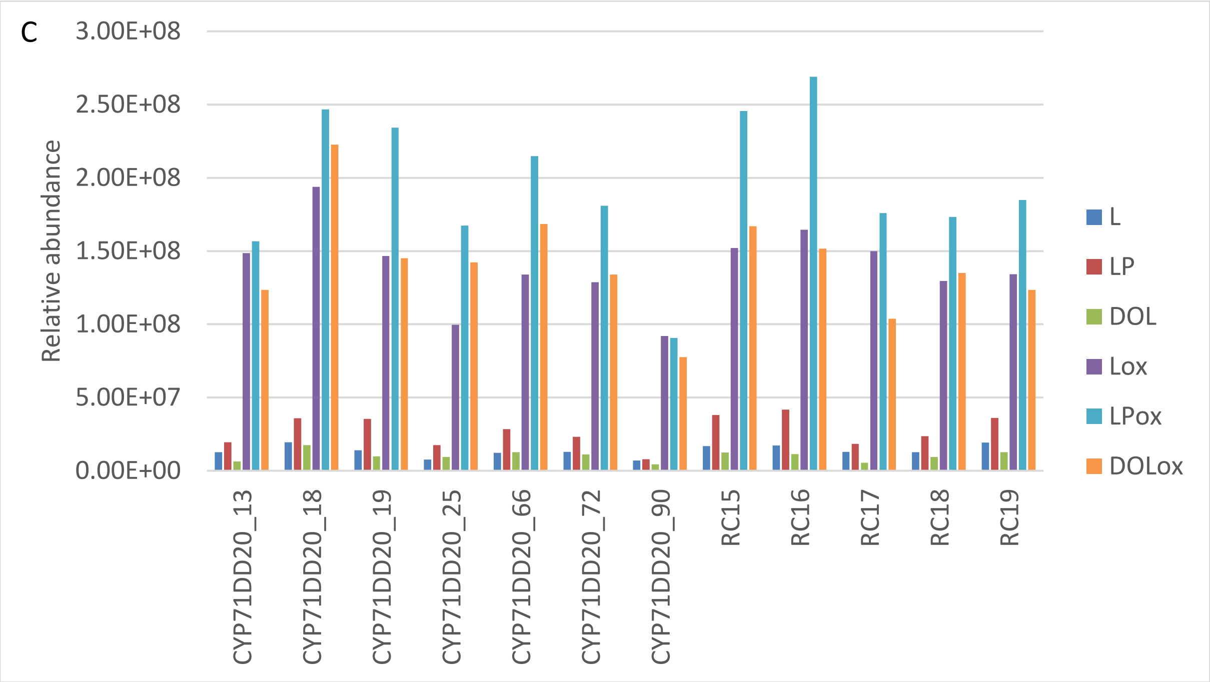
C
Relative abundance
3.00E+08
2.50E+08
2.00E+08
1.50E+08
1.00E+08
5.00E+07
0.00E+00
CYP71DD20_13
CYP71DD20_18
CYP71DD20_19
CYP71DD20_25
CYP71DD20_66
CYP71DD20_72
CYP71DD20_90
RC15
RC16
RC17
RC18
RC19
L
LP
DOL
Lox
LPox
DOLox

Figure S5: Mass spectra of lactucin and 11*β*,13-dihydrolactucin produced in microsomal assays. The mass spectra are shown for Peak 1 – 11*β*,13-dihydrolactucin, and Peak 2 – lactucin, compared to the mass spectra of authentic standards of 11*β*,13-dihydrolactucin and lactucin.

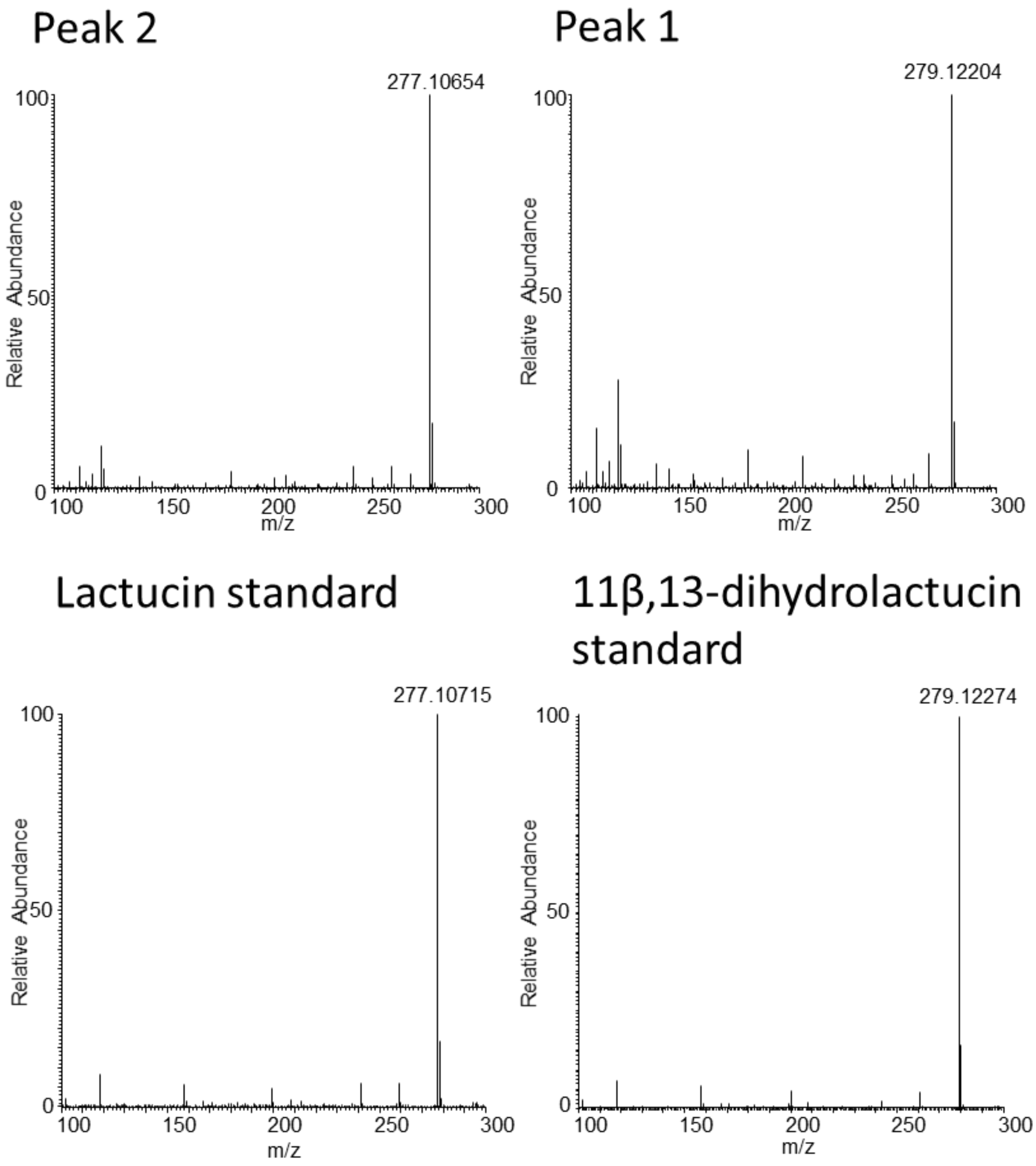

Figure S6: Terpene profiling of taproots of genome edited chicory lines with mutations in *CYP71DD35* and *CYP71DD20* by LC-Orbitrap-FTMS. Peak areas of 6 main sesquiterpene lactones are shown for A) Genome edited chicory lines of gene *CYP71DD35* and B) Genome edited chicory lines of gene *CYP71DD20*. Terpene profiles were compared to regeneration control lines (RC). L - lactucin, LP - lactucopicrin, DOL - 8-deoxylactucin, Lox – lactucin-15-oxalate, DOLox - 8-deoxylactucin-15-oxalate, LPox – lactucopicrin-15-oxalate. Mean and standard deviation of three biological replicates are shown for the genome edited plants and for six biological replicates for regeneration control lines.

A

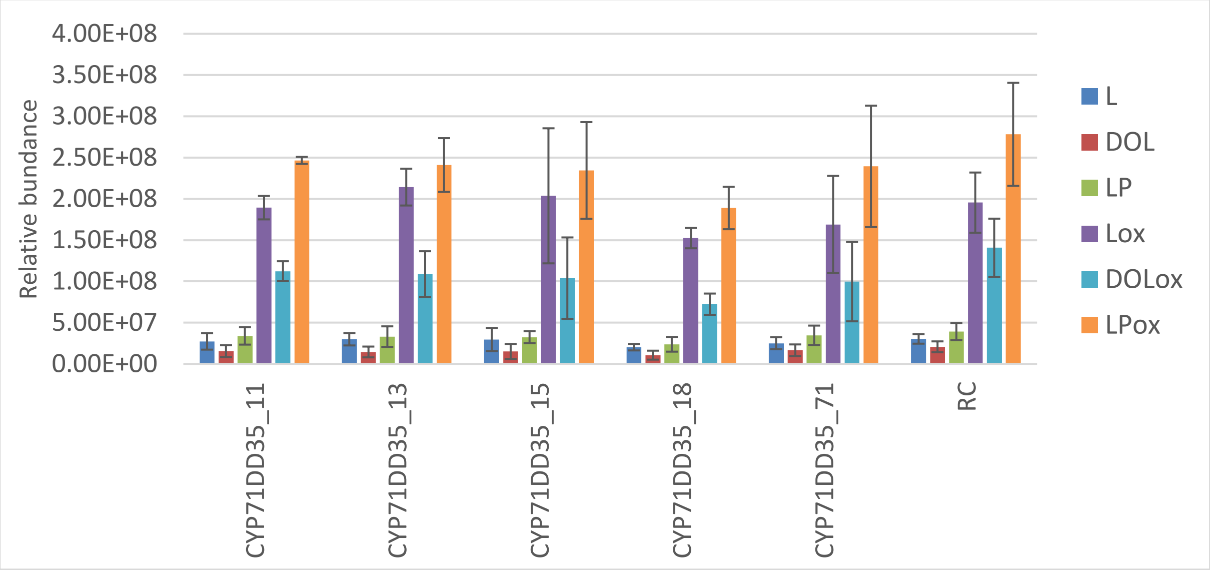

B

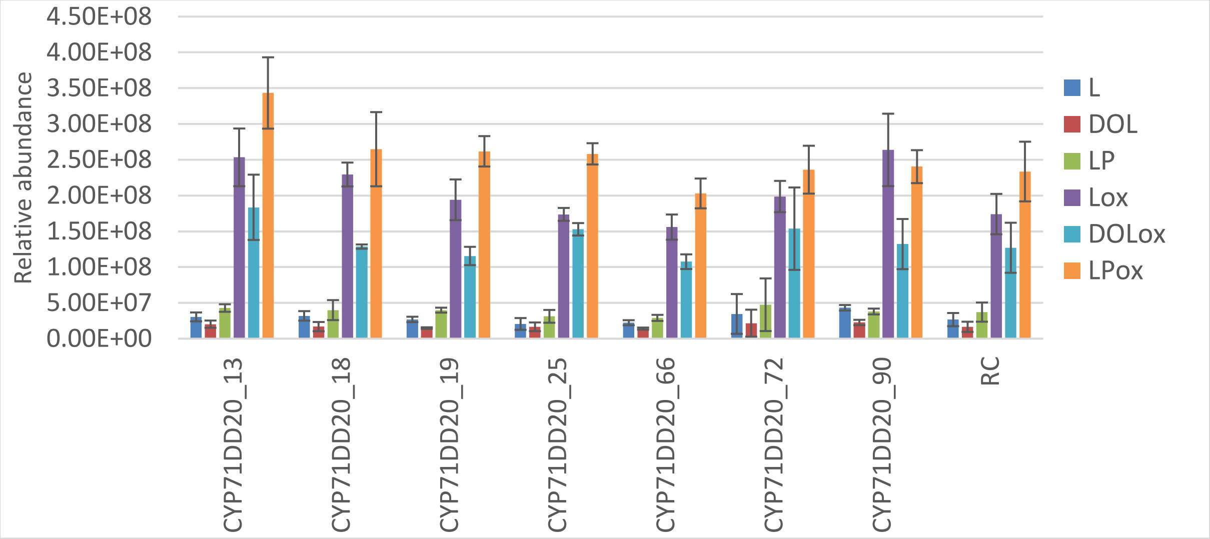

Table S1: Primers and guide RNAs used in this study

Name	Sequence	Target gene	Purpose
CYP71DD20_gRNP	CTATGGTTAATCTAGACGAG	*CYP71DD20*	Guide RNA, RNP based genome editing
CYP71DD35_gRNP	ACGGTATCCATCAAACCATA	*CYP71DD35*	Guide RNA, RNP based genome editing
CYP71DD33_g9	AACCTTCACCAAGTACTGCT	*CYP71DD33*	Guide RNA, plasmid based genome editing
CYP71DD33_g13	TCGTCCAATAAACTTCCCCC	*CYP71DD33*	Guide RNA, plasmid based genome editing
CYP71DD33_g21	TACCTAGCAGTACTTGGTGA	*CYP71DD33*	Guide RNA, plasmid based genome editing
CYP71DD33_g23	TGTCTGGGAAAGCTTCCATA	*CYP71DD33*	Guide RNA, plasmid based genome editing
CYP71DD33_Fw1	CCCAAACGCTCTTTCCTTCATGGTTT	*CYP71DD33*	Genotyping
CYP71DD33Rev2	CATTTGTTGCATACCATCACAAGTGCT	*CYP71DD33*	Genotyping
CYP71DD35_Fw1	AGCACCCCATCCATTAAGC	*CYP71DD35*	Genotyping
CYP71DD35_Rw1	ACTAACCACACCCTCCATCTC	*CYP71DD35*	Genotyping
CYP71DD20_Fw1	ATGGAAGGAGATGCGAAAGG	*CYP71DD20*	Genotyping
CYP71DD20_Rw1	GGAAACCGAACCGCAAAGAGA	*CYP71DD20*	Genotyping
aCas9 Fw2	CTACCAGACTCAAGAGAACCGCTAG	*Cas9*	Cas9 detection
aCas9 Rev2	GTGGTGCTCATCGTATCTCTTGATCA	*Cas9*	Cas9 detection
CYP71DD35 nF	ACTCTTTTCCCTACACGACGCTCTTCCGATCTAGGCTTCCACTAATTGGGAA	*CYP71DD35*	Nested sequencing primer
CYP71DD35 nR	TGGAGTTCAGACGTGTGCTCTTCCGATCTAATGAAGAAGCATAGCTG	*CYP71DD35*	Nested sequencing primer
CYP71DD20 nF	ACTCTTTTCCCTACACGACGCTCTTCCGATCTATACCGAGGTCAAGGGCATG	*CYP71DD20*	Nested sequencing primer
CYP71DD20 nR	TGGAGTTCAGACGTGTGCTCTTCCGATCTCACAATGTCTGTAACGAGAT	*CYP71DD20*	Nested sequencing primer

Table S2: Detailed genotyping of mutations in regenerated genome edited chicory lines

Chicory line	Allele 1	Allele 2	Mutation type
CYP71DD33_20	40 bp deletion (g13-g21)<br>9 bp deletion (g23)	55 bp deletion (g21-g23)	biallelic
CYP71DD33_24	9 bp deletion (g13)<br>12bp deletion (g21)<br>13bp deletion (g23)	89 bp deletion (g13-g21)	biallelic
CYP71DD33_26	17 bp deletion (g21)<br>4 bp deletion (g23)	-	homozygous
CYP71DD33_53	125 bp insertion (g21)	43 bp deletion (g21-g23)	biallelic
CYP71DD33_57	102 bp deletion (g13)	-	homozygous
CYP71DD33_64	A insertion (g13)<br>50 bp deletion (g23)	-	homozygous
CYP71DD33_78	2 bp deletion (g13)<br>1 bp deletion (g21)<br>7 bp deletion (g23)	90 bp deletion (g13-g23)	biallelic
CYP71DD33_85	91 bp deletion (g13-g23)	39 bp deletion (g13-g21)	biallelic
CYP71DD35_11	5 bp deletion	WT	heterozygous
CYP71DD35_13	8 bp deletion	WT	heterozygous
CYP71DD35_15	8 bp deletion	2 bp deletion	biallelic
CYP71DD35_18	5 bp deletion	1 bp insertion	bialellic
CYP71DD35_71	5 bp deletion	1 bp deletion	bialellic
CYP71DD20_13	14 bp deletion	5 bp deletion	bialellic
CYP71DD20_18	5 bp deletion	7 bp deletion	bialellic
CYP71DD20_19	8 bp deletion	9 bp deletion	bialellic
CYP71DD20_25	6 bp deletion	7 bp deletion	bialellic
CYP71DD20_66	1 bp insertion	8 bp deletion	bialellic
CYP71DD20_72	16 bp deletion	WT	heterozygous
CYP71DD20_90	6 bp deletion	8 bp deletion	bialellic

Table S3: Off-target genome editing by guide targeting *CYP71DD20*

Line	Target gene<br>No SNPs	Off-target 1<br>2 SNPs	Off-target 2<br>2/1 SNPs	Off-target 3<br>2 SNPs	Off-target 4<br>1 SNP	Off-target 5<br>1 SNP
CYP71DD20_13	-14/-5	WT/WT	WT/WT	WT/WT	WT/WT	-25/-25
CYP71DD20_18	-5/-7	WT/WT	WT/-5	WT/WT	WT/WT	+1/+1
CYP71DD20_19	-8/-9	WT/WT	WT/WT	WT/WT	WT/WT	+1/+1
CYP71DD20_25	-6/-7	WT/WT	WT/WT	WT/WT	-5/-5	WT/WT
CYP71DD20_66	+1/-8	WT/WT	WT/WT	WT/WT	WT/WT	-6/-6
CYP71DD20_72	WT/-16	WT/WT	WT/WT	WT/WT	+1/+1	WT/WT
CYP71DD20_90	-6/-8	WT/WT	WT/-5	WT/WT	WT/WT	WT/WT
